# Electrowetting limits electrochemical CO_2_ reduction in carbon-free gas diffusion electrodes†

**DOI:** 10.1039/d3ya00285c

**Published:** 2023-09-28

**Authors:** Lorenz M. Baumgartner, Andrey Goryachev, Christel I. Koopman, David Franzen, Barbara Ellendorff, Thomas Turek, David A. Vermaas

**Affiliations:** a Department of Chemical Engineering, Delft University of Technology Netherlands D.A.Vermaas@tudelft.nl; b Institute for Chemical and Electrochemical Process Engineering, Technical University Clausthal Germany

## Abstract

CO_2_ electrolysis might be a key process to utilize intermittent renewable electricity for the sustainable production of hydrocarbon chemicals without relying on fossil fuels. Commonly used carbon-based gas diffusion electrodes (GDEs) enable high Faradaic efficiencies for the desired carbon products at high current densities, but have limited stability. In this study, we explore the adaption of a carbon-free GDE from a Chlor-alkali electrolysis process as a cathode for gas-fed CO_2_ electrolysis. We determine the impact of electrowetting on the electrochemical performance by analyzing the Faradaic efficiency for CO at industrially relevant current density. The characterization of used GDEs with X-ray photoelectron spectroscopy (XPS) and X-Ray diffraction (XRD) reveals a potential-dependent degradation, which can be explained through chemical polytetrafluorethylene (PTFE) degradation and/or physical erosion of PTFE through the restructuring of the silver surface. Our results further suggest that electrowetting-induced flooding lets the Faradaic efficiency for CO drop below 40% after only 30 min of electrolysis. We conclude that the effect of electrowetting has to be managed more carefully before the investigated carbon-free GDEs can compete with carbon-based GDEs as cathodes for CO_2_ electrolysis. Further, not only the conductive phase (such as carbon), but also the binder (such as PTFE), should be carefully selected for stable CO_2_ reduction.

## Abstract 2

Electrowetting leads to flooding of silver-based, carbon-free gas diffusion electrodes for CO_2_ electrolysis.

## Graphical abstract

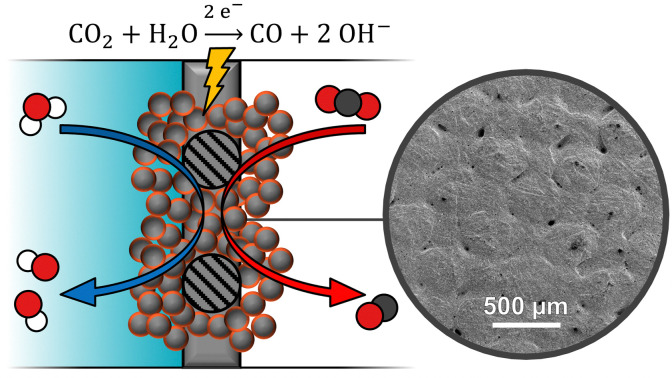

## Introduction

Electrochemical CO_2_ reduction (CO_2_R) could utilize excess wind and solar power to allow the sustainable production of hydrocarbon chemicals, fuels, or plastics.^1,2^ If combined with CO_2_ capture from the atmosphere^3,4^ or the ocean,^5^ this process could be independent of fossil feedstocks and contribute to the goal of the Glasgow Climate Pact to limit the increase in global temperature to 1.5 °C.^6^

Captured CO_2_ can be converted electrochemically with a CO_2_ electrolyzer. Depending on the catalyst employed at the cathode, CO_2_R can yield a range of chemical products (Ag: CO; Sn: HCOOH; Cu: C_2_H_4_). Despite suitable catalysts being available, CO_2_R can still suffer from poor Faradaic efficiency due to mass transfer limitations. If the supply of CO_2_ to the catalyst cannot match the applied current density, the competing hydrogen evolution reaction (HER) takes place.^7^ While the production of CO on Ag has the highest maturity among the different CO_2_R routes, the process for CO production still has to be further optimized to meet a number of key industrial criteria:^8^

•Current density, *j*: −200 to −500 mA cm^−2^

•Faradaic efficiency, FE_CO_: >95%

•Catalyst activity: 100 A g^−1^

•Stability: >10 000 h

High *j*_CO_2_R_ has become feasible through the introduction of gas diffusion electrodes (GDEs). By avoiding mass transfer limitations imposed by the limited solubility and diffusivity of CO_2_ in aqueous electrolytes,^9–11^ GDEs suppress the unwanted HER and allow high FE for C_1_ products^12–14^ and C_2_ products.^15–17^ Typically, GDEs for CO_2_R consist of a catalyst layer (CL), coated on top of a gas diffusion layer (GDL). The CL provides a reaction interface, at which gaseous reactants get in contact with the catalyst surface and the electrolyte. Catalysts are typically employed in the form of nanoparticles^18^ with typical loadings of 1 mg cm^−2^,^19^ which allows specific catalyst activities of >200 A g^−1^. The GDL is typically treated with polytetrafluoroethylene (PTFE) to prevent electrolyte intrusion and ensure free pore space for gas transport. The majority of studies utilizes commercial, carbon-based GDL materials adapted from hydrogen fuel cell research.^20,21^

While the first three criteria for industrial application are within reach with current materials, only a limited long-term stability has been reported for carbon-based GDEs. CO_2_ electrolyzers with membrane electrode assembly (MEA) have achieved *j*_CO_2_R_ values of ≥−200 mA cm^−2^ and FE_CO_ ≥90% for up to 1000 h (Table 1).^14^ More commonly, however, much shorter lifetimes than this are reported because insufficient hydration management leads to rapid electrode drying^22^ or carbonate salt formation in the gas channels.^23–25^ Even shorter lifetimes have been reported for CO_2_ electrolyzers with a catholyte buffer (Table 1). Similar problems with GDE stability are also known from fuel cell applications, in which carbon and PTFE can degrade significantly after 100 h of operation at target current densities, which leads to a loss of hydrophobicity. In turn, the pore network of the GDL becomes more flooded with liquid, which reduces the performance by inhibiting the gas transport.^26^

**Table 1:** Stability of GDEs in electrolyzers. Carbon-based GDEs typically consist of a catalyst layer deposited on a commercial gas diffusion layer (catalyst/GDL). In MEA reactors, a membrane separates the GDE from the electrolyte. The carbon-free GDE from Covestro (oxygen depolarized cathode) consists of Ag particles and PTFE on a Ag mesh support.^27,28^ The current density is *j*. The reaction temperature is *T*

Process	Gas diffusion electrode	*j* (mA cm^−2^)	*T* (°C)	Electrolyte	Lifetime (h)
Carbon-based GDEs: direct electrolyte contact					
CO_2_R: CO	Ag + Nafion/ELAT LT1400 W cloth^29^	−185	20	1 M KHCO_3_	>120
CO_2_R: CO	Ag + Nafion/SGL 39BC^30^	−190	20	1 M KHCO_3_	≤20
CO_2_R: HCOOH	Sn + carbon + PTFE/SGL 35BC^31^	−200	20	1 M KHCO_3_	>5
CO_2_R: C_2_H_4_	Cu/Freudenberg (unspecified)^16^	−320	20	7 M KOH	<1
CO_2_R: CO	Ag/Freudenberg H23C6^32^	−196	20	1 M KOH	<0.25

Carbon-based GDEs: membrane electrode assembly (MEA)					
CO_2_R: CO	Ag + Sustainion/SGL 35BC^14^	−200	20	10 mM KHCO_3_	>1000
CO_2_R: CO	Ag + Nafion/carbon felt^22^	−100	20	1 M NaOH	<2

Oxygen depolarized cathodes (ODCs): direct electrolyte contact					
ORR	Ag + carbon + PTFE/Ag mesh^33^	−300	80	32 wt% NaOH	>28 800
CO_2_R: CO	Ag + PTFE/Ag mesh (Covestro)^34^	−300	30	0.4 M K_2_SO_4_ + 1.5 M KHCO_3_	>1200
CO_2_R: CO	Ag + PTFE/Ag mesh (Covestro)^35^	−150	20	0.4 M K_2_SO_4_	>840

The lifetime of carbon-based GDEs in CO_2_ electrolyzers depends on the chemical stability of the GDL substrate. As chemical degradation reduces the hydrophobicity of the pore network, electrolyte breakthrough occurs at lower differential pressure between liquid and gas phase,^36^ which limits the flow-by regime in scaled-up electrolyzers.^29^ The reported lifetime of carbon-based GDEs differs between materials types (Table 1). Nonwoven GDLs from Freudenberg exhibit especially short lifetimes of less than 1 h (Table 1).^16,30,32^ Yang *et al.* demonstrated the flooding of a Freudenberg GDL started when the cathode potential was set below −0.65 V *vs.* RHE. X-ray photoelectron spectroscopy (XPS) measurements indicated a degradation of the CFS, which showed a reduction of C–F bonds and an increase in oxygen content.^37^ In contrast, carbon papers manufactured by SGL seem to be more stable (Table 1). For example, we recently demonstrated the operation of a SGL carbon paper at –190 mA cm^−2^ for 20 h until flooding occurred. Post-electrolysis characterization revealed that the static contact angle of the CFS had dropped from initially 149° to 128° after electrolysis.^30^ A woven carbon cloth from ELAT showed a very promising performance, as it was stable for at least 120 h and allowed more than 50% FE_CO_ despite flooding (Table 1).^29^

The adoption of oxygen depolarized cathodes (ODC) for the CO_2_R process might help avoid the limitations of carbon-based GDLs altogether.^38^ ODCs are silver-containing GDEs, which have been employed in industrial chlor-alkali electrolysis for many years. Typically, they consist of a current collector mesh with a porous layer of Ag and PTFE, which allows O_2_ transfer to reaction zone. There, the oxygen reduction reaction (ORR) is carried out at 80–90 °C with 30–35 wt% NaOH electrolytes.^39–41^ ODCs have been shown to be stable for ten thousands of hours in these harsh chemical conditions (Table 1).^33^ Early ODCs used carbon particles as a catalyst support, which limited their long-term stability (>10 000 h) because carbon is susceptible to degradation.^39,40^

Modern ODCs have a carbon-free composition to enable a higher long-term stability.^39^ For example, the commercial, carbon-free ODC from Covestro consists of Ag particles (92–98 wt%) and PTFE (2–8 wt%) on a Ag mesh support.^27,28^ Unfortunately, the GDE from Covestro is proprietary, which means that limited public characterization data is available.^27,28^ In our previous work, we developed our own carbon-free ODC, which has been optimized for ORR,^42,43^ and characterized it with advanced imaging techniques (*e.g.*, X-ray tomography, *operando* X-ray radiography).^44,45^

As silver is a common catalyst for CO_2_R, carbon-free ODCs that only use silver as the electrically conductive medium could be an interesting alternative to commonly used carbon-based GDEs. So far, only a limited number of publications has investigated the application of these carbon-free GDEs for CO_2_R. For example, the commercial ODC from Covestro has been successfully employed for up to 1200 h in CO_2_ electrolyzers with flowing catholyte (Table 1).^34,35^ The performance of these electrodes is limited by CO_2_ mass transfer at higher *j*, as FE_CO_ falls below 80% beyond current densities of −300 mA cm^−2^.^46^ Studying the carbon-free GDEs developed in our previous work,^42,43^ Hoffmann *et al.* used *operando* synchrotron imaging to reveal the detrimental effect of electrolyte intrusion into the pore network of the GDE on the CO_2_ mass transfer to the catalyst. This previous study shows that the electrolyte intrusion into the cathode, or flooding, depends on the applied potential and the PTFE content. They achieved the highest current density of −300 mA cm^−2^ at a PTFE content of 3 wt% (97 wt% Ag) when testing for ≤50 min.^47^

The flooding of the GDE with electrolyte is detrimental to the CO_2_ performance because the intruding electrolyte lowers the effective diffusivity of the pore network by displacing the gas phase.^48^ At open circuit potential, the flooding resistance of GDEs depends on material properties, such as the pore structure or wettability. For example, high flooding resistance can be achieved with small, hydrophobic pores.^48^ This can be achieved by the addition of PTFE, which decreases the average pore diameter by filling gaps between the silver particles in the GDE.^42,43^ Further, the addition of PTFE increases the contact angle, which increases the hydrophobicity of the internal pore walls.^42,43^

GDE flooding also depends on the electrowetting effect,^44,45,49^ which describes the physical phenomenon of surfaces becoming more hydrophilic when an electrical potential is applied. This has important implications for the flooding behavior of porous GDEs because the internal contact angles of the pore network are reduced as the electrode is charged during electrolysis. Under these conditions, the electrolyte can infiltrate the pore network and hinder gas diffusion.^45,47^

In this work, we study the CO_2_R performance of carbon-free GDEs by assessing the chemical stability and analyzing the Faradaic efficiency for CO at industrially relevant current density (−200 mA cm^−2^). We conducted experiments with a carbon-free GDE (97 wt% Ag, 3 wt% PTFE) and a typical carbon-based GDE made with a SGL 39BC substrate to allow a direct comparison between these two electrodes types. We assess the impact of electrowetting on the CO_2_R performance of carbon-free GDEs. In addition, the used electrodes were characterized with XPS and X-ray diffraction (XRD) to measure changes to the chemical composition. Based on these assessments, we discuss the feasibility of adopting carbon-free GDEs for CO_2_ electrolysis.

## Experimental methods

The electrode preparation, physical characterization, and electrochemical experiments are described in more detail in the ESI.†

### Electrode preparation

#### Preparation of the carbon-free GDE

The carbon-free GDEs were prepared by spray deposition.^43^ A suspension was mixed from 30 g Ag particles, 50 g of a solution with 1 wt% hydroxyethyl methyl cellulose, 40 g water to adjust the viscosity, and 1.5 g of a dispersion with 59 wt% PTFE. A silver gauze was used as a current collector. It was fixed in a frame and placed on a heating plate (100 °C) to facilitate the drying process. Then, the suspension was deposited onto the gauze in 80 homogeneous layers with an airbrush. The composition of the deposited layer was 97 wt% Ag and 3 wt% PTFE. The target Ag loading was 160 mg cm^−2^. The coated sample was hot-pressed at 130 °C and 15 MPa for 5 min. Subsequently, we placed the GDE in an air oven at 330 °C for 15 min to form pores by burning out methylcellulose and to sinter the Ag and PTFE.

#### Preparation of the carbon-based GDE

The carbon-based GDEs were prepared by depositing a silver catalyst layer on a commercial carbon-based GDL with a spray deposition process.^30^ The ink suspension for the catalyst layer was mixed from 33 mg Ag nanopowder, 2.1 mL water, 2.1 mL propan-2-ol, and 180 μL of a 5 wt% Nafion D-521 dispersion. The mixture was homogenized in a sonication bath for 30 min. The GDL substrate (SGL 39 BC, SGL Carbon) was placed on a heating table (130 °C) equipped with a 2D-motorized stage. The ink was evenly sprayed onto the MPL side with an airbrush. The target composition of the deposited catalyst layer was 80 wt% Ag and 20 wt% Nafion. The target Ag loading was 1 mg cm^−2^.

#### Electrode characterization

The static contact angle was measured with the sessile drop method.^50^ We recorded images of 10 μL water droplets at five different locations of the sample. The contact angle was extracted with the image processing software ImageJ (Fig. S2, ESI†).^51^

The liquid breakthrough pressure (at open circuit potential) was measured by placing the sample in a transparent flow cell and pumping water into the liquid compartment (Fig. S1, ESI†). By closing off the liquid outlet, water was forced to break through the porous sample. We recorded the differential pressure at which the first droplet appeared on the gas side (Fig. S3, ESI†).

The convective mass transfer was analyzed by studying the CO_2_ permeability. After installing the sample in a flow cell, we passed CO_2_ through the material at different flow rates and measured the pressure drop (Fig. S4, ESI†). By plotting the flow rate against the pressure drop, we derived the permeability constant from the slope according to Darcy's law.^52^

The diffusive mass transfer was evaluated by determining the limiting overall O_2_ mass transfer coefficient. We use this metric as a proxy for the CO_2_ mass transfer coefficient.^30^ The GDE was placed in a flow cell with 6 M KOH in the liquid compartment. We flowed air through the gas compartment and measured the gas pressure (Fig. S5, ESI†). We carried out a linear sweep voltammetry (LSV) scan for the ORR. The value of *K*_O_2__ was derived from the plateau current density (Fig. S6, ESI†).

Scanning electron microscopy (SEM) was carried out with a JSM-6010LA microscope (JEOL, Japan) at an acceleration voltage of 5 kV. The images were recorded with a secondary electron imaging (SEI) and a back-scattered electron composition (BEC) detector.

X-ray diffraction (XRD) patterns were obtained using a Bruker D8 Advance diffractometer equipped with a Co anode (*λ*(Kα) = 1.7889 Å, 35 kV, 40 mA) and a Lynxeye position-sensitive detector. The diffractograms were acquired in the Bragg–Brentano geometry with a step size of 0.02° and an acquisition time of 4 s. A motorised varied-divergent slit (V6) and constant rotation of the holder (30 RPM) were applied. The diffractograms were processed in DiffracSuite.EVA (v.5.1) software. The Scherrer equation was used to evaluate changes to the crystallite size.^53^

X-ray photoelectron spectroscopy (XPS) measurements were carried out on a K-Alpha XPS spectrometer (Thermo Scientific), equipped with a small-spot (400 μm) monochromatic X-ray source (Al Kα = 1486.6 eV). Core level spectra were recorded with a pass energy of 50 eV. Low energy Ar^+^ ions were used to compensate surface charging. CasaXPS software was used for data processing. The spectra were normalized on the C 1s binding energy (BE) of the adventitious carbon (284.8 eV). XPS depth profiles were recorded on the same spectrometer. Ar^+^ sputtering was done at 3 kV with time steps of 60 s.

#### CO_2_ electrolysis procedure

**Fig. 1:**
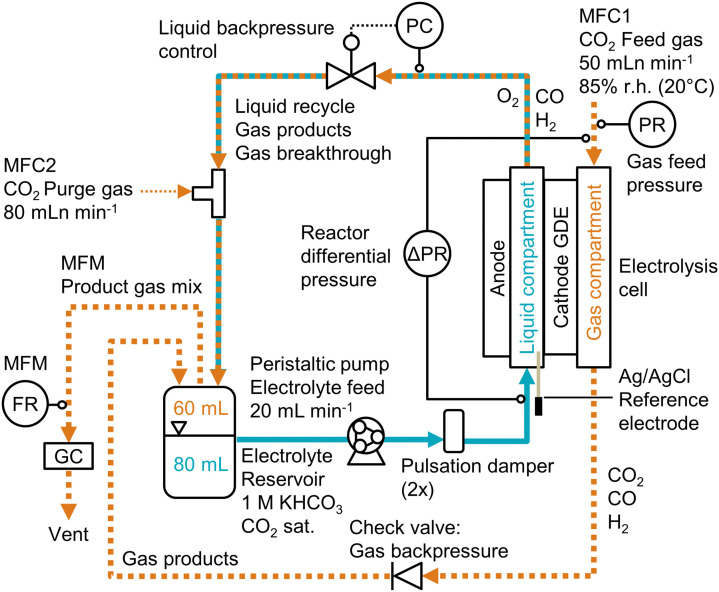
Process flow diagram for CO_2_ electrolysis setup. The backpressure of the electrolyte stream was controlled (PC) before it was mixed with the purge gas and recirculated. The differential pressure, Δ*p*, across the GDE was measured between the catholyte and gas compartment (ΔPR). The Faradaic efficiency was determined by recording the product gas flow rate (FR) with a mass flow meter (MFM) and analyzing the gas composition by gas chromatography (GC).

The CO_2_ reduction performance was determined with an automated setup (Fig. 1). The cathode GDEs were installed in a membraneless flow cell (Fig. S1, ESI†). A mass flow controller (MFC1) supplied the CO_2_ feed at a flow rate of 50 mLn min^−1^. The CO_2_ was humidified to 85% relative humidity at 20 °C in two bubble columns and fed into the gas compartment (Fig. S8, ESI†). The backpressure of the gas was set by the cracking pressure of a check valve. The 1 M KHCO_3_ electrolyte was recirculated with a peristaltic pump. The liquid backpressure was controlled with an electronic control valve to set the flow-by regime at the GDE. After passing through the flow cell, the liquid stream was mixed with a purge gas, which was supplied at a flow rate of 80 mLn min^−1^, in order to facilitate the transfer of the product gases into the headspace of the electrolyte reservoir. From there, the product gas mixture passed to the gas chromatography (GC) system. The flow rate was measured with a mass flow meter (MFM). We performed a series of current density steps ranging from −10 to −200 mA cm^−2^. For each step, we carried out at least two GC injections to determine the Faradaic efficiency for CO and H_2_ (Fig. S11, ESI†).

The cathode potential was recorded with an Ag/AgCl micro-reference electrode and corrected for the ohmic resistance of the electrolyte. The full cell potentials are measured as well, but are relatively high (2–8 V) due to the thick electrolyte (22 mm) channel. Because the reference electrode was placed close to the cathode, the cathode potential is insensitive to the cell configuration. Hence, unless indicated otherwise, all reported potentials are cathode potentials, given in the reversible hydrogen electrode (RHE) scale. All recorded process parameters, such as cell potentials, pressures, or GC data, are listed in the accompanying excel file of the ESI.†

## Results & discussion

The prepared GDE samples were characterized and their CO_2_ reduction performance assessed with galvanostatic measurements. The chemical stability of the GDEs was assessed by additional characterization after the electrolysis experiments. Supplementary results and the numerical values of all plotted data are included in the ESI.†

### Electrode characterization

**Fig. 2:**
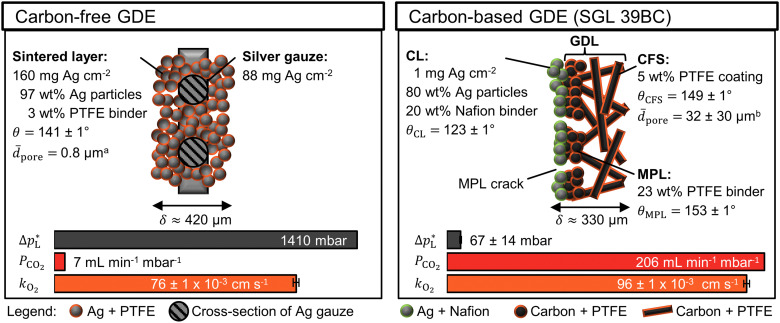
Physical properties of carbon-free GDE and carbon-based GDE (GDL: SGL 39BC carbon paper). The static contact angle, *θ*, ± its standard error were calculated from five or more measurements. ^a^The average pore diameter, *d̄*_pore_, was obtained from Franzen *et al.*^43^ for the carbon-free GDE. ^b^The *d̄*_pore_ of the SGL 39 BC's carbon fiber substrate (CFS) was obtained from Parikh *et al.*^57^ The total thickness of the GDE is *δ*. The liquid breakthrough pressure is 
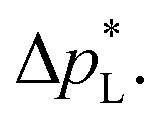
 The CO_2_ permeability constant is *P*_CO_2__ The limiting overall O_2_ mass transfer coefficient is, *k*_O_2__, (proxy for CO_2_ mass transfer). Its error bars indicate the standard deviation of the limiting currents. More detailed characterization data are available in the ESI.†

The carbon-free GDE consists of the sintered Ag-PTFE composite forming a uniform layer around the current collector mesh (Fig. S12, ESI†).^43^ Both sides of the GDE exhibit a static contact angle of *θ* = 141° at open circuit (Fig. 2). The material is more hydrophobic compared to a flat silver surface (*θ*_Ag_ = 95°)^54^ because of the 3 wt% PTFE binder (*θ*_PTFE_ = 122°).^55^ In addition, the sintered GDE has a rough surface which allows gas pockets to enhance the contact angle according to the Cassie–Baxter model.^50^ An overview of all measured contact angles is given in Table S2 (ESI†).

The carbon-based GDE is made up of the catalyst layer (CL), which is coated on top of the GDL (Fig. 2). The GDL has two components: the microporous layer (MPL) and the carbon fiber substrate (CFS). The MPL consists of carbon black and PTFE particles, which give it a high contact angle (*θ*_MPL_ = 153°). The CFS is comprised of graphitized carbon fibers, which have been impregnated with PTFE (*θ*_CFS_ = 149°). We note that our static contact angles have a mostly qualitative meaning as they do not capture the effects of contact angle hysteresis on rough surfaces or the internal contact angle,^56^ which are critical to quantify the flooding behavior of the pore network.

The carbon-free GDE has a more than 20× higher liquid breakthrough pressure at open circuit, 
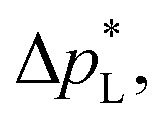
 than the carbon-based GDE (Fig. 2: 1410 *vs.* 67 mbar). This high resistance against liquid intrusion is due to the unimodal pore structure with a small average diameter of *d̄*_pore_ = 0.8 μm,^43^ In contrast, the carbon-based GDL exhibits a much lower 
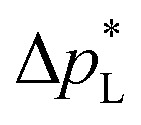
. As the MPL commonly features large cracks due to the manufacturing process (Fig. S14, ESI†), this layers adds little flooding resistance despite the high *θ*_MPL_ and small pore size.^30,36^ Instead, 
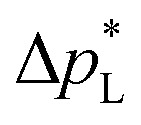
 is mostly determined by the properties of the CFS, whose pores are much larger (32 μm)^57^ and consequently exhibit a much lower capillary pressure.

The high 
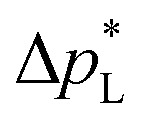
 of the carbon-free GDE is very promising for scale-up because it determines how well the GDE could maintain the separation of gas and liquid phase at a large scale. For illustration, a 
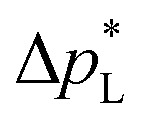
 of 1.4 bar corresponds to resisting the hydrostatic pressure of an aqueous electrolyte in a 14 m tall cell, which is an order of magnitude larger than the height of commercial cells for alkaline electrolysis (1–2 m)^58^ or chlor-alkali electrolysis (1–1.5 m).^59,60^ However, the flooding resistance also has to be assessed under operating conditions because electrowetting can reduce the hydrophobicity and thereby decrease 
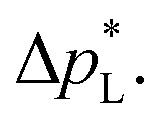
^29,36^

The mass transfer between the gas bulk and the reaction zone can occur through convection and/or diffusion. The carbon-free GDE has a 30× smaller capacity for convective mass transfer compared to the carbon-based GDE, which is quantified with the CO_2_ permeability constant, *P*_CO_2__ (Fig. 2: 7 *vs.* 206 mL min^−1^ mbar^−1^). According to the Hagen–Poiseuille equation (*F*_G,pore_ ∝ *d*_pore_^4^), the flow rate through a pore scales with the fourth power of the diameter. We can therefore expect the carbon-free GDE's small pores to result in a lower *P*_CO_2__. In contrast, the carbon-based GDE has relatively large CFS pores and large cracks allowing a large portion of the gas flow to bypass the small pores of the MPL. As proposed in a previous study, we can expect diffusion to be the dominating mass transfer mechanism in the flow-by regime.^30^ For this reason, the lower *P*_CO_2__ of the carbon-free GDE should not hamper the CO_2_ reduction performance significantly.

The carbon-free GDE has a 20% lower capacity for diffusive mass transfer, *K*_O_2__ (Fig. 2: 76 *vs.* 96 × 10^−3^ cm s^−1^). We quantified this capacity with the limiting overall O_2_ mass transfer coefficient, *k*_O_2__, which serves as a proxy for the experimentally less accessible limiting CO_2_ mass transfer coefficient.^30^ The lower diffusive mass transport for the carbon-free GDE is explained by its higher thickness and smaller pore size, which lead to a longer, more tortuous diffusion pathway compared to its carbon-based counterpart. It may be beneficial to reduce the thickness of the GDE by using a thinner current collector gauze and depositing fewer layers of silver and PTFE.

The silver loading of the carbon-free GDE is almost 250× higher compared to the carbon-based GDE (Fig. 2: 248 mg Ag cm^−2^*vs.*1 mg Ag cm^−2^). As silver is a relatively expensive metal, we can expect this higher loading to increase the capital expenditure of the electrode significantly. Therefore, we test the CO_2_R performance to assess if the carbon-free GDE the higher material costs with a higher productivity and/or lifetime.

### CO_2_ electrolysis: electrowetting inhibits performance

**Fig. 3:**
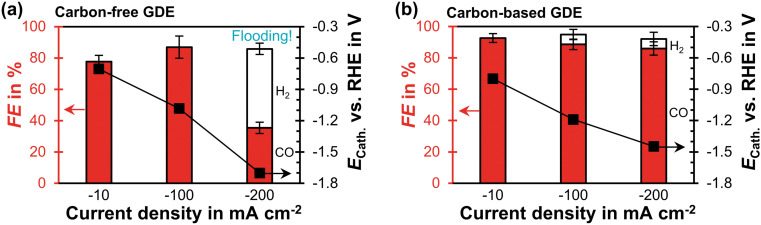
CO_2_ reduction performance in flow-by regime at steady state: the Faradaic efficiency, FE, for CO or H_2_ is plotted as a function of current density on the left *y*-axis. The error bars represent the estimated standard errors. The cathode potential, *E*_Cath_, against the reversible hydrogen electrode (RHE) is plotted on the right *y*-axis. The potential was corrected for the ohmic potential drop between the reference electrode and the cathode. (a) Carbon-free GDE: each data point represents an average calculated from two GC injections. Flooding occurred after 10 min at −200 mA cm^−2^. The two injections were taken 30 min later. (b) Carbon-based GDE: each data point is based on three GC injections. The data is taken from a previous publication.^29^

The carbon-free GDE has a similar CO_2_ reduction performance at −100 mA cm^−2^ as the carbon-based GDE (Fig. 3). At −200 mA cm^−2^ its FE_CO_ becomes significantly lower compared to the carbon-based GDE (35% *vs.* 86%). Based on the relative diffusivities of the characterization results, we would expect the carbon-free GDE to achieve a proportionally lower FE_CO_ of 69%. Because this is not the case, however, we hypothesize that the additional performance drop is caused by the observed electrolyte flooding. Flooding fills empty pores with liquid and decreases the effective diffusivity of the porous GDE. As a consequence, the CO_2_ mass transfer falls below the supply of electrons, so that the HER takes place instead of the CO_2_R. The cathode potential, *E*_Cath_, probably becomes more negative compared to the carbon-based GDE because evolving hydrogen gas bubbles increase the ohmic resistance (−1.4 V compared to −1.7 V *vs.* RHE). We can further investigate this inferior performance by comparing the short-term stability.

**Fig. 4:**
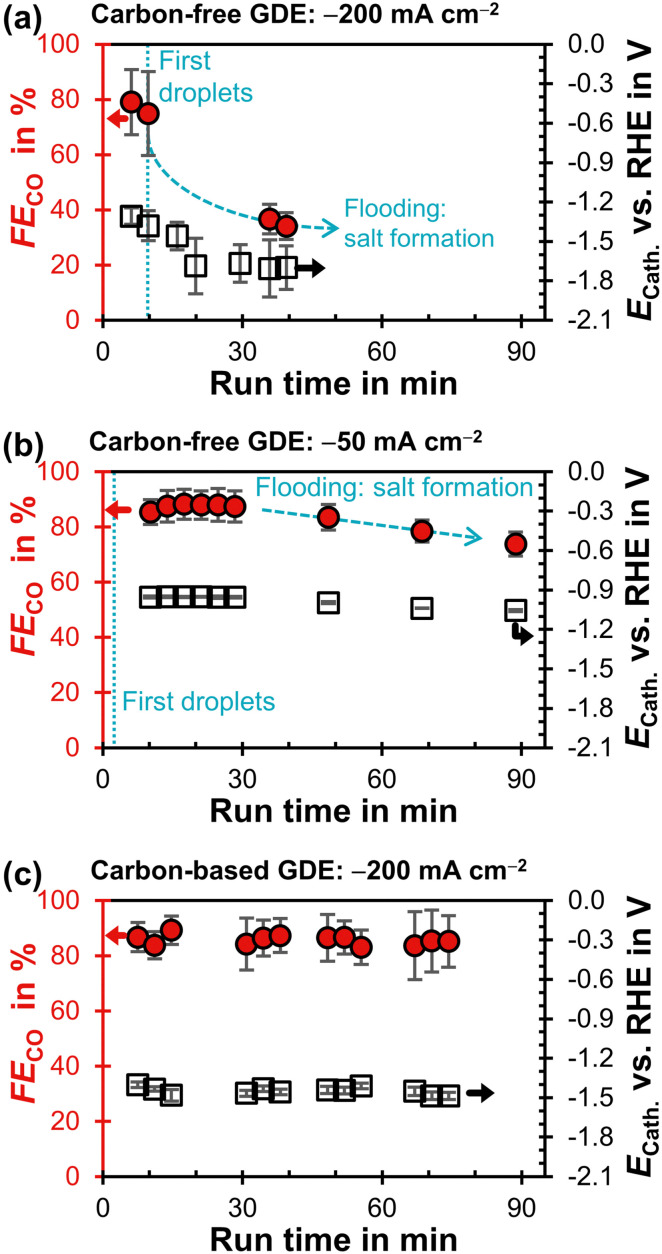
Faradaic efficiency for CO, FE_CO_, as a function of run time after starting the potentiostat. The *iR*-compensated cathode potential, *E*_Cath_, against the reversible hydrogen electrode (RHE) is plotted on the right y-axis. Every data point represents a single GC injection. The error bars represent the estimated standard error. (a) Carbon-free GDE at −200 mA cm^−2^. (b) Carbon-free GDE at −50 mA cm^−2^. (c) Carbon-based GDE (GDL: SGL 39BC) at −200 mA cm^−2^.^29^

The carbon-free GDE does not allow stable CO_2_R at −200 mA cm^−2^ and has a much lower FE_CO_ compared to the carbon-based GDE (Fig. 4a and c). First droplets of electrolyte appear on the gas side of the GDE 10 min after the current density is applied and the cathode potential drops below −1.3 V. This suggests that the initially high flooding resistance is lost due to electrowetting. As the run proceeds, the FE_CO_ declines steadily while the electrolyte droplets increase in size and start to dry out. After a run time of 40 min, (bi)carbonate salts start forming on the surface of the GDE (Fig. S15, ESI†).

We carried out another electrolysis run with the carbon-free GDE at −50 mA cm^−2^ in an attempt to mitigate the electrowetting-induced flooding with a less negative cathode potential (Fig. 4b). FE_CO_ declines more slowly as the initial *E*_Cath_ is less negative compared to the −200 mA cm^−2^ run (−1.0 V compared to −1.3 V). This slower flooding is in agreement with X-ray imaging studies, which report that the speed of electrolyte intrusion is potential-dependent.^45,49^ Ultimately, however, the CO_2_R performance is also not stable at −1.0 V because the flooding leads to salt formation on the gas side.

Our results (Fig. 4) raise the question why electrowetting leads to a more detrimental flooding for the carbon-free than for the carbon-based GDE. Electrowetting spreads an electrolyte more strongly on bare, conductive surfaces (*e.g.*, silver, carbon) than on surfaces that are covered with a dielectric insulator (*e.g.*, PTFE).^45,61^ We hypothesize that the carbon-based GDE's higher PTFE content (MPL: 23 wt%, CFS: 5 wt%) covers bare carbon surfaces more effectively, which leads to a stronger insulation against electrowetting. In contrast, the PTFE in the carbon-free GDE has a lower concentration (3 wt%) and is distributed heterogeneously throughout the pore network.^44,45^ This structure probably allows the electrolyte to transition from a non-wetting Cassie–Baxter state to a wetting Wenzel state, when the potential is decreased sufficiently.^61,62^ This means that the electrolyte does not rest on the dispersed PTFE, but spreads along the bare silver surfaces through electrowetting.^45^

**Fig. 5:**
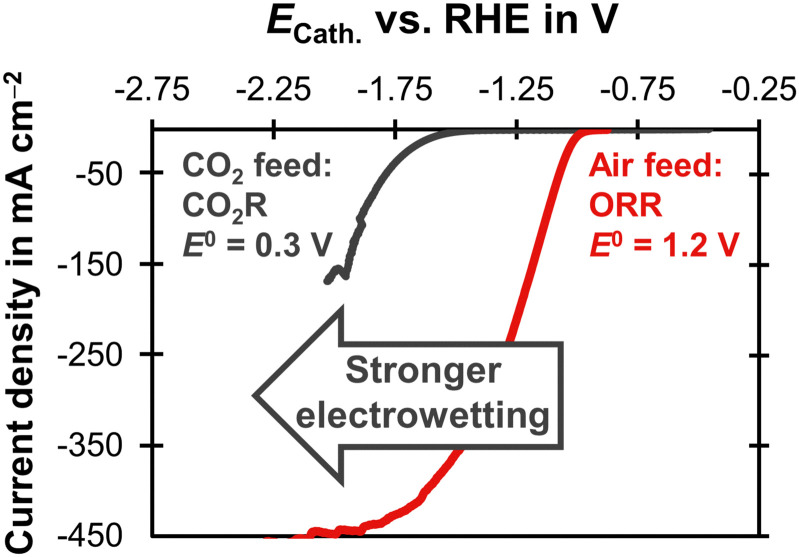
Linear sweep voltammetry scans comparing CO_2_R and ORR on a carbon-based GDE with a Ag loading of 1 mg cm^−2^ on the basis of a SGL 39BC GDL. The current density is plotted as a function of the cathode potential, *E*_Cath_. The potential was corrected for the ohmic potential drop between the reference electrode and the cathode. The experiments were conducted at 20 °C with a scan rate of 20 mV s^−1^. The CO_2_R experiment was carried out in 1 M KHCO_3_ (pH = 7.5) with a CO_2_ gas feed. The equilibrium potential, *E*^0^, was obtained from Jouny *et al.*^63^ The ORR experiment used 6 M KOH (pH = 14.8) and an air gas feed. The *E*^0^ was obtained from Mousallem *et al.*^39^

Electrowetting leads to a poor performance for CO_2_R with the carbon-free GDE (Fig. 4). This is interesting because electrowetting and electrolyte breakthrough also occur during ORR, but they do not seem to have such a detrimental effect for this reaction.^42,49^ We hypothesize that the flooding due to electrowetting is stronger for the CO_2_R because this reaction requires more negative cathode potentials compared to the ORR (Fig. 5). Therefore, the electrolyte saturates the pore network to a higher extent and the supply of CO_2_ is severely limited.

The poor CO_2_R performance of our carbon-free GDEs (3 wt% PTFE) suggests that a different manufacturing approach is required to mitigate the effect of electrowetting and ensure high gas diffusivity under operating conditions. While increasing the PTFE content further, for example to 8 wt% PTFE, significantly reduces the intrusion of electrolyte,^47^ this is not a viable strategy because the additional PTFE also prevents the wetting of catalyst sites and reduces the amount of open pore space available for gas diffusion.^43^ Perhaps this problem could be solved by^43,47^ fabricating the GDE from two layers with varying PTFE content. The (I) reaction layer should have a relatively low PTFE content (≤3 wt%) and would provide a large wetted catalyst surface for the CO_2_R reaction. The (II) diffusion layer should have a relatively high PTFE content (≥6 wt%) to make it more resilient against electrowetting and ensure the transport of CO_2_ from the gas bulk to the reaction layer. Perhaps it is also possible to selectively increase the PTFE content of the GDE's gas-facing side by introducing an additional PTFE coating step to the manufacturing process.

### Post electrolysis characterization: PTFE degradation occurs at high overpotential

In addition to reversible electrowetting, the GDE can also experience a permanent loss of hydrophobicity through chemical reactions, which also has a negative impact on performance and is detrimental to the long-term stability. After CO_2_ electrolysis, we rinsed the electrodes with water to remove carbonate salts. We then measured the static contact angle and analyzed chemical changes with SEM, XPS, and XRD.

The XPS analysis of the GDE surface shows no clear change in Ag oxidation state (Fig. S20, ESI†), in line with the applied cathodic potentials. The XRD diffractograms of fresh and used GDEs show a single cubic phase of Ag^0^ with no variation of crystalline parameters (Fig. S17, ESI†). These results indicate that the surface did not oxidize and that the crystallite size of Ag particles did not change by CO_2_R.

**Fig. 6:**
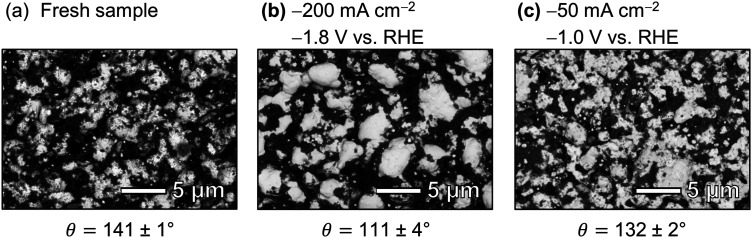
SEM images and static contact angle, *θ*, of carbon-free GDEs: elemental contrast images were recorded with the BEC detector of the SEM (Light grey domains: Ag, dark grey domains: PTFE). The average *θ* ± the standard error were determined with the sessile drop method. (a) Fresh GDE sample. (b) After electrolysis at −200 mA cm^−2^ for 84 min with an initial cathode potential of −1.8 V *vs.* RHE (potential stabilized at −1.4 V *vs.* RHE). Additional SEM images are available in Fig. S18 (ESI†).

However, elemental contrast imaging with SEM/BEC suggests that the amount of surface PTFE was reduced by the electrolysis at −200 mA cm^−2^ (Fig. 6). Particularly, the silver backbone of a fresh GDE was covered with finely dispersed PTFE particles, in contrast to the large silver clusters emerged at the surface of the spent sample. The morphology change of spent GDEs was also reflected in a significant reduction of *θ* (Fig. 6: 111° *vs.* 141°), which is in line with the observed removal of hydrophobic PTFE domains from the electrode surface. We hypothesize that the removal of PTFE is caused by chemical degradation and/or physical erosion due to restructuring of the silver surface.

The XPS survey reveals that the loss of hydrophobicity is accompanied by a change in surface chemistry. The electrolysis reduced the fluorine concentration from 58 at% *vs.* 40 at%, while the carbon concentration increased from 36 at% *vs.* 49 at% (Table 2). Further, the chemical state of carbon changes as the fraction of C–R bonds increases at the cost of C–F bonds (Table 2). These results support the hypothesis that PTFE undergoes reductive electrochemical degradation. According to Shapoval *et al.*,^64^ this mechanism takes place below the cathode potential threshold of −1.3 V (Fig. S23, ESI†). In its course, fluoride is eliminated from the PTFE polymer chain^64^ and carbonaceous decomposition products are left behind.^64,65^

**Table 2:** XPS measurements of carbon-free GDEs: assessment of elemental composition and the chemical state of carbon (CF_2_: C–F bonds in PTFE; CO_*x*_: COR, CO, COOR; CR: C–H or C–C bonds. C–X: all carbon bonds). (a) Fresh GDE sample. (b) After electrolysis at −200 mA cm^−2^ for 84 min with an initial cathode potential of −1.8 V *vs.* RHE (stabilized at −1.4 V *vs.* RHE). (c) After electrolysis at −50 mA cm^−2^ for 89 min with cathode potential of −1.0 V *vs.* RHE. The analyzed area was facing the electrolyte during electrolysis

Sample	(a) Fresh sample	(b) −200 mA cm^−2^ −1.8 V *vs.* RHE	(c) −50 mA cm^−2^ −1.0 V *vs.* RHE
Elemental surface composition			
F	58 at%	40 at%	51 at%
C	36 at%	49 at%	35 at%
Ag	4 at%	4 at%	6 at%
O	1 at%	6 at%	4 at%

Relative fraction of carbon bonds			
CF_2_/C–X	84%	45%	76%
CO_*x*_/C–X	4%	7%	4%
CR/C–X	13%	48%	20%

The slight increase in the atomic concentration of oxygen and the fraction of CO_*x*_ bonds (Table 2) is probably caused by residual potassium (bi-)carbonate salts. The presence of these salts was confirmed by the small amount of potassium in the XPS signal of the used sample (Table S6 and Fig. S21, ESI†).

**Fig. 7:**
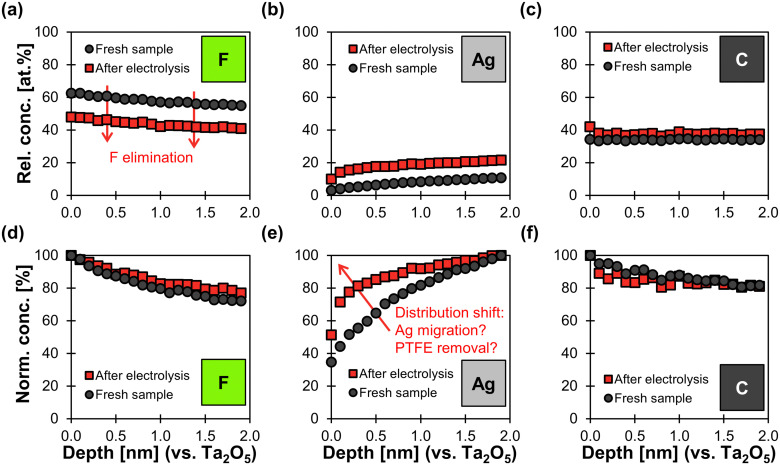
XPS depth profiles of fresh carbon-free GDE and spent sample after electrolysis. Electrolysis was performed at −200 mA cm^−2^ for 84 min with an initial cathode potential of −1.8 V *vs.* RHE (stabilized at −1.4 V *vs.* RHE). The *x*-axis shows the depth profile calibrated against a Ta_2_O_5_ standard sputtered with Ar^+^ ions. (a)–(c): the *y*-axis shows the relative atomic concentrations of F, Ag, and C (other elements were not considered in this analysis mode). This means that the three elements together make up 100 at% in this plot. (d)–(f): The *y*-axis shows the normalized concentrations for each elements along the profile. This means that the depth with the highest atomic concentration along the profile determines the 100% value in the normalized concentration plot.

XPS depth profile analysis shows a uniform reduction of 14 at% in fluorine content along the surface profiled after electrolysis (Fig. 7a). As a consequence, the relative concentration of Ag increases by an average of 11 at% along the depth profile. The relative carbon content increases only slightly by 4 at% along the profile (Fig. 7c). These findings agree with the hypothesis that electrochemical degradation eliminates fluoride from the PTFE polymer chain and leaves behind carbonaceous degradation products.^64,65^

In addition, the normalized Ag distribution of the used sample is shifted towards the surface (Fig. 7e), which might be due to the migration of Ag and/or the removal of PTFE close to the top surface. This phenomenon could be explained by a potential-induced restructuring of the Ag surface,^27,65^ which might cause the PTFE to loose adhesion and fall off. Such a restructuring would be in agreement with the smoother Ag surfaces observed in the SEM images (Fig. 6). We can therefore conclude that physical erosion of PTFE due to Ag restructuring probably takes place in parallel to chemical degradation.

The carbon-free GDE that was operated at −50 mA cm^−2^ and a cathode potential of −1.0 V also underwent chemical changes. The XPS analysis shows that the surface concentration of fluorine is lower than for the fresh sample (Table 2: 58 at% *vs.* 51 at%). The fraction of CF_2_ bonds drops from 84% to 76% (Table 2). These changes to the surface chemistry are accompanied by a reduction of *θ* from 141 ± 1° to 132 ± 2° compared to the fresh sample (Fig. 6). However, the SEM images suggest that the silver matrix remains covered with dispersed PTFE (Fig. 6). The XPS depth profiles vary little compared to a fresh sample (Fig. S22, ESI†). These results show that the degradation of the GDE is less significant at −1.0 V (−50 mA cm^−2^) compared to the GDE that operating at −1.8 V (−200 mA cm^−2^).

It is noteworthy that the degradation of PTFE also seems to take place below the reported potential threshold of −1.3 V *vs.* RHE.^64^ A possible explanation for this inconsistency might be that the difference in solvent affects the elimination of fluoride from the polymer chain. Shapoval *et al.*^64^ studied PTFE degradation in anhydrous DMF, while our electrolyte was aqueous KHCO_3_. Other interesting questions for future study are how fast the degradation occurs over time and if the mechanism only depends on the cathode potential or also on the total charge passed through the GDE.

Our carbon-free GDEs underwent chemical degradation after less than 1.5 h of CO_2_ electrolysis. Long-term CO_2_ electrolysis was successfully performed for more than 1200 h by Haas *et al.* with the carbon-free GDEs from Covestro.^34^ These electrodes were characterized after electrolysis, which revealed a restructuring of the silver surface. Further, Raman microscopy showed a shift in signal from PTFE to carbon,^27^ which is probably a sign of PTFE degradation at the surface. This example suggests that carbon-free GDEs can tolerate some chemical degradation during long-term CO_2_ electrolysis. A possible explanation for this tolerance might be that the removal and degradation of PTFE occurs primarily close to the surface while leaving the internal pores less affected. Nonetheless, it is critical that the sintered silver backbone retains its morphology and pore size to ensure stable long-term operation.

The investigated carbon-free GDEs are not a feasible cathode for CO_2_ electrolysis. We can expect carbon-free GDEs to require higher capital expenditure because the substitution of carbon with silver increases the loading by two orders of magnitude (*e.g.*Fig. 2 carbon-free: 248 mg Ag cm^−2^*vs.* carbon-based: 1 mg Ag cm^−2^). To recover these additional costs, carbon-free GDEs require a higher productivity and/or lifetime compared to carbon-based GDEs. At a *j* of −200 mA cm^−2^, the finely dispersed Ag catalyst in carbon-based GDEs achieves a specific productivity of 200 A g^−1^. This already satisfies the productivity criterion of 100 A g^−1^ proposed by Masel *et al.*^8^ In contrast, our carbon-free GDEs only reach a silver catalyst productivity of 1 A g^−1^ at the same *j*. To make matters worse, our carbon-free GDEs achieved a FE_CO_ 40% due to the effects of electrowetting.^34,46^

## Conclusion

We have investigated the adoption of a carbon-free GDE for electrochemical CO_2_ reduction (CO_2_R), which was originally developed for the oxygen reduction reaction (ORR) in the chlor-alkali process. The GDE experienced a poor FE_CO_ (≤40%) at cathode potentials beyond −1.0 V *vs.* RHE (−50 mA cm^−2^) due to physical electrowetting. Electrowetting reduces the hydrophobicity of the porous GDE, which blocks gas diffusion paths by electrolyte flooding and (bi)carbonate salt formation. Exposing the GDE to potentials of −1.8 V *vs.* RHE (−200 mA cm^−2^) for <90 min resulted in a partial degradation and removal of PTFE from the GDE. This might suggest that a poor chemical stability limits the long-term stability of these electrodes, but it was not possible to deconvolute the effect of degradation from the effect of electrowetting on the FE_CO_. Compared with commonly used carbon-based GDEs, the investigated carbon-free GDE exhibit a worse production rate of CO and did not allow steady state operation. We conclude that the effect of electrowetting has to managed more carefully before the investigated carbon-free GDE can compete with carbon-based GDEs as cathode for CO_2_ electrolysis.

## Conflicts of interest

There are no conflicts to declare.

## Supplementary Material

YA-002-D3YA00285C-s001

YA-002-D3YA00285C-s002
