# Supplementary material for: Electrowetting limits electrochemical CO2 reduction in carbon-free gas diffusion electrodes
Source: Energy Adv. 2023 Sep 28;2(11):1893–904. doi: 10.1039/d3ya00285c (PMC10634457; doi:10.1039/d3ya00285c)
Supplement: YA-002-D3YA00285C-s001 [file YA-002-D3YA00285C-s001.pdf]

Electronic Supplementary Material (ESI) for Energy Advances.
This journal is © The Royal Society of Chemistry 2023

SUPPORTING INFORMATION

Electrowetting Limits Electrochemical CO_2 Reduction in carbon-free Gas Diffusion Electrodes

Lorenz M. Baumgartner,[a] Andrey Goryachev,[a] Christel I. Koopman,[a] David Franzen,[b] Barbara Ellendorff,[b] Thomas Turek,[b] David A. Vermaas*,[a]

[a] Department of Chemical Engineering, Delft University of Technology, Netherlands

[b] Institute for Chemical and Electrochemical Process Engineering, Technical University Clausthal, Germany

Email: D.A.Vermaas@tudelft.nl

Supporting Information Content:

- 22 Pages
- 9 Tables
- 23 Figures

Contents

1. General information 2

2. Methods 2

2.1 GDE preparation 2

2.1.1 Preparation of the carbon-free GDEs 2

2.1.2 Preparation of the carbon-based GDEs 2

2.1.3 Sample overview 3

2.2. Assembly of membraneless CO_2 electrolysis cell 4

2.3 Electrode characterization 5

2.3.1 Scanning electron microscope (SEM): Microstructure investigation 5

2.3.2 Sessile drop contact angle: Wettability assessment 5

2.3.3 Liquid breakthrough pressure: Flooding resistance 6

2.3.4 CO_2 Permeability constant: Convective mass transfer 6

2.3.5 Limiting overall O_2 mass transfer coefficient: Diffusive mass transfer 7

2.4 CO_2 electrolysis experiments 9

2.4.1 Engineering of the CO_2 electrolysis setup 9

2.4.2 Experimental timeline for CO_2 reduction performance with current density steps 13

3. Supplementary results and discussion 14

3.1 Microstructure investigation (SEM) 14

3.2. CO_2 reduction performance with current density steps: Flooding and salt formation 15

3.3. Post electrolysis characterization: Assessment of chemical stability 16

3.3.1 XRD: No changes to silver bulk composition 16

3.3.2 SEM: PTFE surface coverage changes at high cathode overpotential 17

3.3.3. XPS: Degradation and removal of PTFE 18

References 22

1. General information

De-ionized water was used for all experiments. Detailed experimental results are available in the accompanying **Excel file** of the supporting information.

2. Methods

2.1 GDE preparation

We prepared three carbon-free GDEs samples and one carbon-based GDE sample for all experiments.

2.1.1 Preparation of the carbon-free GDEs

The carbon-free GDEs were prepared by spray deposition.[1] The components of the ink suspension were added together in the following order:

- 30 g Ag particles (SF9ED, Ferro GmbH)
- 50 g methyl cellulose solution with 1 wt% hydroxyethyl methyl cellulose (WALOCEL™ MKX 70000 PP 01)
- 40 g water to adjust the viscosity
- 1.5 g PTFE dispersion (TF 5060GZ, 3 M™ Dyneon™: 59 wt% PTFE, 8 wt% surfactant)

A silver gauze was used as current collector (40936 Silver gauze, 80 mesh, 115 µm diameter wire, 99.9% metal basis, Alfa Aesar). The area weight of the gauze was 88 mg Ag cm^{-2}. We fixed the current collector in a frame and placed it on a heating plate (100 °C) to facilitate the drying process. Then, the suspension was deposited onto the gauze in 80 homogeneous layers using an airbrush (Evolution, 0.6 mm pin hole, Harder & Steenbeck). The composition of the deposited layer was 97 wt% Ag and 3 wt% PTFE. The target Ag loading was 160 mg cm^{-1}. We hot-pressed the coated sample at 130 °C and 15 MPa for 5 min (LaboPress P200S, Vogt, Germany). Subsequently, the GDE was placed in an air oven at 330 °C for 15 min to form pores by burning out methylcellulose and to sinter the Ag and PTFE.

2.1.2 Preparation of the carbon-based GDEs

The carbon-based GDEs were prepared by depositing a catalyst layer on a commercial carbon-based GDL with a spray deposition process.[2] We mixed the ink for the catalyst layer in the following order:

- 33 mg Ag nanopowder (Aerodynamic particle size: 20 – 40 nm, 99.9% metal basis, Alfa Aesar)
- 2.1 mL water
- 2.1 mL propan-2-ol
- 180 µL of Nafion D-521 dispersion (5 wt%, Alfa Aesar)

The target composition of the deposited catalyst layer was 80 wt% Ag and 20 wt% Nafion. The Nafion binder content of 20 wt% was selected to match the optimized content determined by Duarte *et al.*.[3] The target Ag loading was 1 mg cm^{-1}. This common catalyst loading was selected to simplify the comparison with other studies.[4] The concentration of solids (Ag + Nafion) in the ink was 0.01 g mL^{-1}. Note that we used an excess of ink to compensate for the loss of 30% ink during the deposition process. We homogenized the ink in a sonication bath for 30 min (USC500TH, VWR). We cut the GDL (SGL 39BC, SGL Carbon) to a size of 3.5 cm x 3 cm, dried it for 10 min at 120 °C, and weighed it in an airtight container (Kartell 034600 Polypropylene Weighing Bottles – 50 mL, Fisher Scientific). The sample was then covered with a 3 cm x 3 cm PTFE mask and fixed to the heating plate (130 °C) of the 2D-motorized stage. We sprayed the ink evenly onto the MPL side with an airbrush (Paasche TG3, Airbrush Services Almere, Netherlands). The sample was dried at 120 °C for 10 min and weighed after the deposition to determine the Ag loading.

2.1.3 Sample overview

Table S1 summarizes the different samples used for the experiments of this publication. The carbon-based GDE samples are identical with the sample from our previous work.[2, 5]

Table S1: Sample overview for carbon-free and carbon-based GDEs. The total thickness of the carbon-free GDEs was determined with a thickness gauge. The total thickness of the carbon-based GDEs is based on the manufacturer data and our own estimation of the catalyst layer thickness (3.5 µm).[2]

[redacted]					
Ag 114-1	Silver	254	419	2, S3, S4	$\theta, \Delta p_{\mathrm{L}}^{*}, P_{\mathrm{CO_2}}$
Ag 114-2	Silver	236	390	3 a, 4 a, S15	FE_{CO}
Ag 114-3	Silver	249	413	2, 6, 7, S16, S17, S18, S20 – S22	$k_{\mathrm{O_2}}, FE_{\mathrm{CO}}, \theta$
Ag 178-1	Silver	269	416	S18, S22	FE_{CO}, θ
45	Carbon	1.2	330	2, 5	$k_{\mathrm{O_2}}, \theta$
47	Carbon	1.3	330	3 b, 4 c	FE_{CO}

2.2. Assembly of membraneless CO_2 electrolysis cell

The GDE samples were installed in the membraneless, 2-compartment flow cell (**Figure S1**).[2]

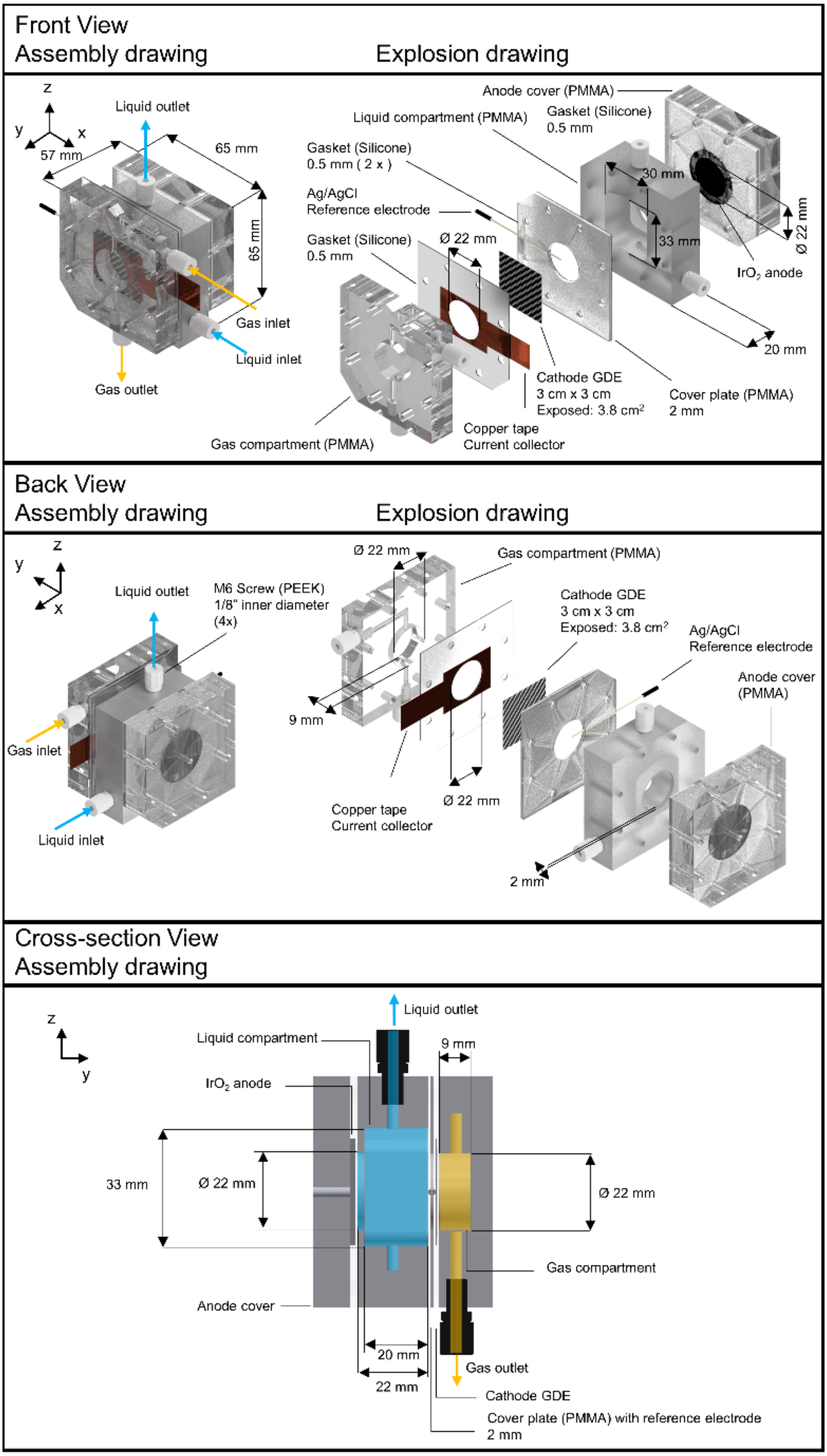

Figure S1: Schematic of the 2-compartment flow cell. The cell body is made of transparent poly methyl methacrylate (PMMA). The screws at the inlet were made of poly ether ether ketone (PEEK).[2]

2.3 Electrode characterization

The silver- and carbon-based GDEs were characterized with various methods before and after electrolysis.

2.3.1 Scanning electron microscope (SEM): Microstructure investigation

The GDE microstructure was visualized with a JSM-6010LA SEM (JEOL, Japan). The morphology was investigated with a secondary electron imaging (SEI) detector at an acceleration voltage of 5 kV and an electron beam spot size of 30. The elemental contrast was imaged with a backscattered electron composition (BEC) detector operated at 5 kV and a spot size of 35.

2.3.2 Sessile drop contact angle: Wettability assessment

We described the relevant wetting theory and experimental method in more detail in a previous publication.[2] The wettability of (external) surfaces was quantified with the sessile drop method. We recorded images of a 10 µL water droplet at five different locations of the sample (**Figure S2 a** and **b**). The static contact was extracted with the image processing software ImageJ and the Contact angle plugin. The contact angle was determined by marking the outline of the droplet and the intersection with the solid interface manually. An ellipse was fit to the outline of the droplet (**Figure S2 c** and **d**). The left and right ellipse angle, $\theta_{\mathrm{E,L}}$ and $\theta_{\mathrm{E,R}}$, were determined from the intersection of the ellipse tangents with the line of the solid interface. They are used to calculate the average ellipse angle, θ_{E}. The static contact angle, θ, is calculated with $\theta = 180 - \theta_{\mathrm{E}}$. The averaged θ for each sample is listed in **Table S2**. The complete list of contact angles can be found in the **accompanying Excel file**.

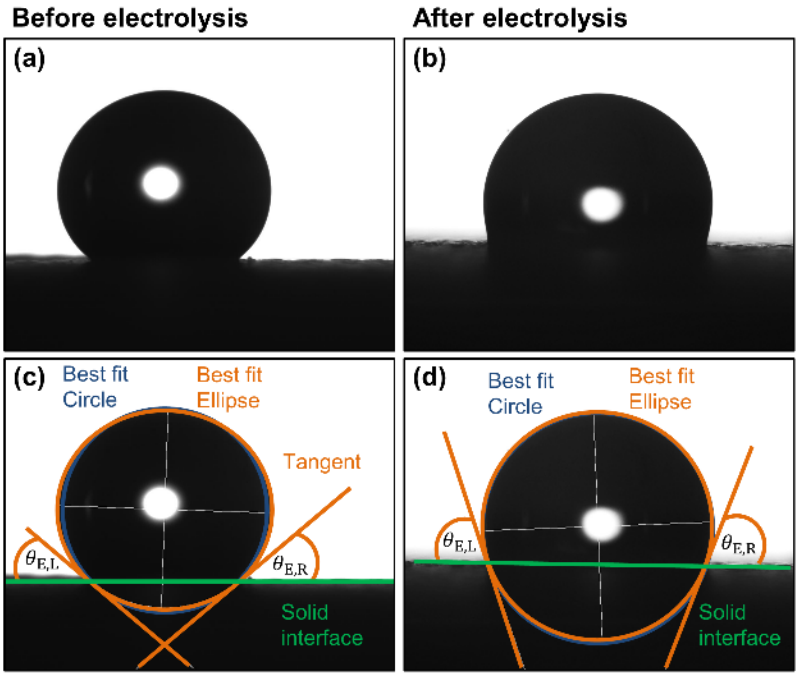

Figure S2: Data analysis example for static contact angle, θ, with sessile drop technique. **(a)** and **(b)**: Raw data images. **(c)** and **(d)**: Corresponding data processing images generated with ImageJ and the contact angle plugin. The angles $\theta_{\mathrm{E,L}}$ and $\theta_{\mathrm{E,R}}$ arise between the intersection of the tangents of the ellipse and the solid interface line. They are used to calculate the average ellipse angle, θ_{E}. The value of θ is calculated with $\theta = 180 - \theta_{\mathrm{E}}$.

Table S2: Static contact angle, θ, average ± the corresponding standard error for at least five measurement locations on silver- and carbon-based GDEs.

**Before electrolysis**	
Carbon-free GDE (Sample Ag 114-1)	141 ± 4.3°
Carbon-based GDE (Sample 45) - CFS	149 ± 1.1°
Carbon-based GDE (Sample 45) - MPL	153 ± 0.9°
Carbon-based GDE (Sample 45) - CL	123 ± 1.5°
**After electrolysis**	
Carbon-free GDE (Sample Ag 114-3: –200 mA cm^{-2})	111 ± 4.3°
Carbon-free GDE (Sample Ag 178-1: –50 mA cm^{-2})	132 ± 1.9°

2.3.3 Liquid breakthrough pressure: Flooding resistance

To measure the liquid breakthrough pressure at open circuit, Δp_L^*, the sample was installed in the 2-compartment flow cell (**Figure S1**). We closed off the outlet of the liquid compartment (**Figure S3 a)** and filled the compartment with water at a flow rate of 1 mL min^{-1}. We determined Δp_L^* by recording the differential pressure between the gas and liquid compartment when the first water droplet appeared at the surface of the sample (**Figure S3 b)**. For the carbon-based GDE, two samples of uncoated SGL 39BC gas diffusion layers were used to determine the average Δp_L^*. The data for this material was reported in our previous publication.[2]

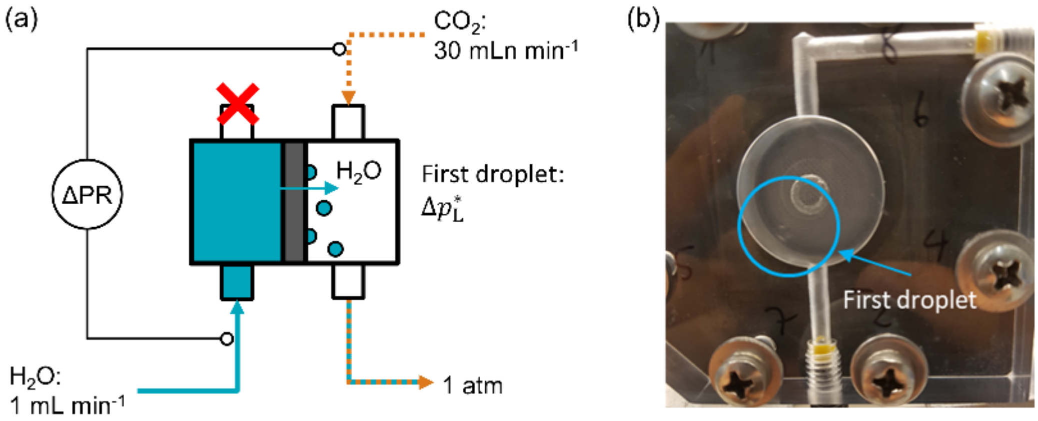

Figure S3: Flooding resistance (at open circuit): **(a)** Flow chart for liquid breakthrough pressure, Δp_L^*, measurement. **(b)** Example image of liquid droplet appearing on gas side of sample.

2.3.4 CO_2 Permeability constant: Convective mass transfer

To measure the CO_2 permeability constant, $P_{\mathrm{CO_2}}$, the sample was installed in the 2-compartment flow cell (**Figure S1**). We supplied CO_2 at different flow rates and recorded the pressure drop across the sample (**Figure S4 a**). The gas was forced through the sample by closing off the outlet of both compartments. The values for $P_{\mathrm{CO_2}}$ were determined from the linear slope of the resulting pressure drop curve **Figure S4 b** according to Darcy's law.[2, 6] We used an uncoated carbon-based GDL for the SGL 39BC sample.[2]

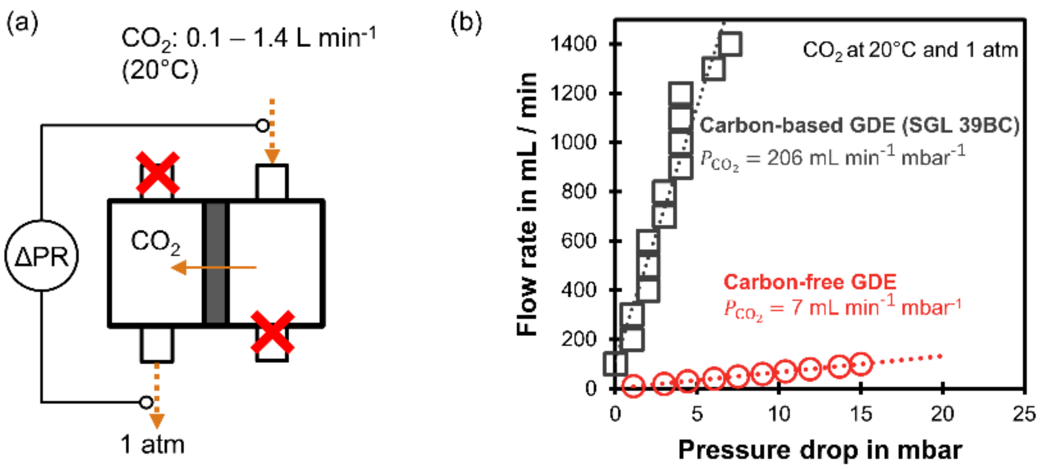

Figure S4: Convective mass transfer capacity: **(a)** Flow chart for measurement of CO_2 permeability constant, P_{CO_2}. **(b)** Resulting pressure drop curves to determine P_{CO_2} from the linear slope.

2.3.5 Limiting overall O_2 mass transfer coefficient: Diffusive mass transfer

We measured the limiting overall O_2 mass transfer coefficient, k_{O_2}, with the electrochemical procedure described in a previous publication.[2] The oxygen from an air feed is reduced to hydroxide ions at the cathode GDE according to the oxygen reduction reaction (ORR) (**Figure S5**). A Nickel plate served as a counter electrode. We used 6 M KOH as the electrolyte due to its high conductivity. We balanced the pressure between the gas and the liquid compartment to achieve a flow-by regime, in which the transfer of O_2 from the gas bulk to the catalyst layer occurs primarily through diffusion. The cathode potential was recorded with a Ag/AgCl micro-reference electrode.

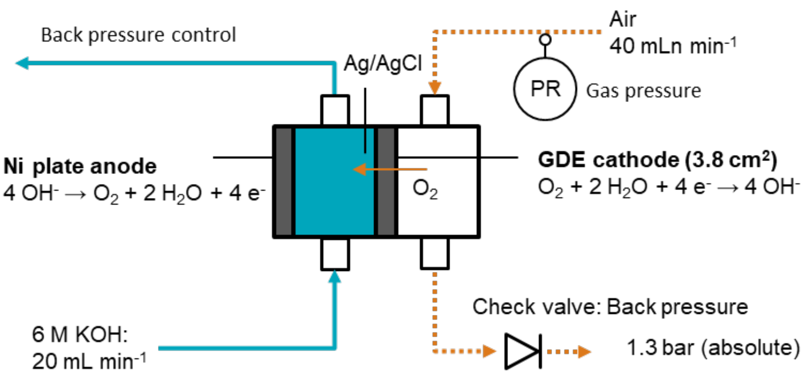

Figure S5: Experimental configuration for limiting oxygen mass transfer measurement. The cathode GDE reduces oxygen at the liquid–catalyst interface to OH^- ions according to the oxygen reduction reaction (ORR). The current is limited by the diffusive mass transfer rate of O_2 from the gas bulk through the GDL to the catalyst layer.[2]

We performed linear sweep voltammetry from 0 V to –2 V vs. SHE with a scan rate of 20 mV s^{-1}. The limiting current density, j_{lim}, was derived from the plateau region of the scan, at which the ORR was limited by oxygen diffusion through the GDE (**Figure S6**). We only used a single scan to determine j_{lim} because the flooding due to electrowetting might influence on consecutive scans.

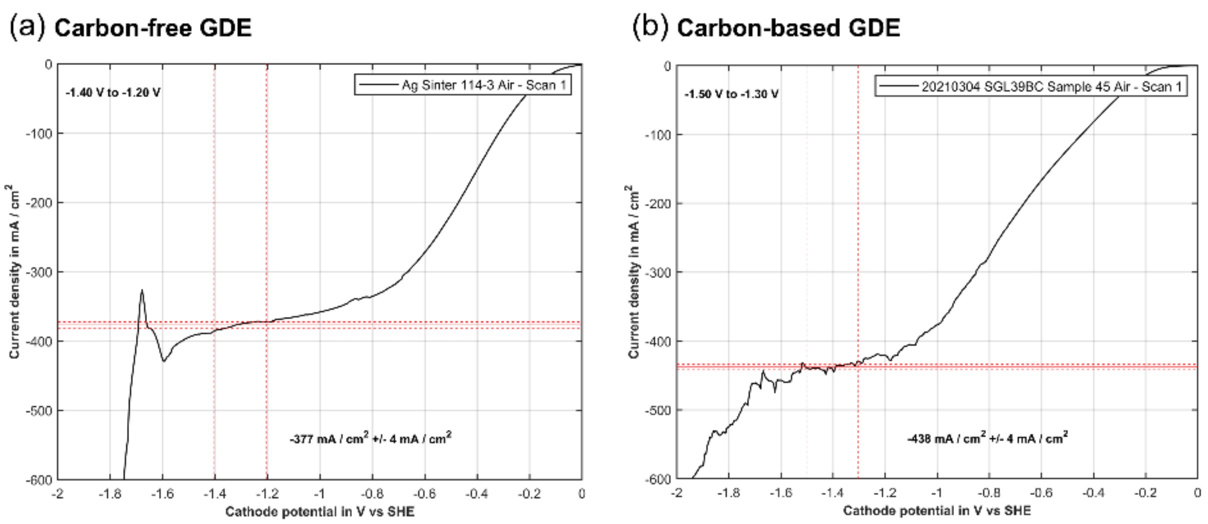

Figure S6: Limiting overall O_2 mass transfer coefficient: linear sweep voltammetry scans to determine the limiting current density plateau. The scan rate was 20 mV s^{-1}. The limiting current density, j_{lim}, ± its standard deviation $\sigma_{j_{\mathrm{lim}}}$ is marked with the red horizontal lines within the manually determined potential window marked with the red vertical lines. **(a)** Carbon-free GDE. **(b)** Carbon-based GDE.

As already described in our previous work,[2] the limiting O_2 molar flux, $\dot{n}_{\mathrm{O_2,lim}}$, in mol cm^{-2} s^{-1} was calculated from j_{lim} with Faraday's law (S1). Faraday's constant is $F = 96485$ C s^{-1} and the number of electrons exchanged in the ORR is $z = 4$.

$$\dot{n}_{\mathrm{O_2,lim}} = \frac{j_{\mathrm{lim}}}{F \cdot z} \tag{S1}$$

We assume that the limiting O_2 flux, $\dot{n}_{\mathrm{O_2,lim}}$, is proportional to the overall O_2 mass transfer coefficient of the GDE, $k_{\mathrm{O_2}}$ in cm s^{-1}, and the O_2 concentration gradient between the bulk of the gas compartment, $C_{\mathrm{O_2,bulk}}$, and the catalyst surface, $C_{\mathrm{O_2,Cat.}}$. We neglected concentration gradients in flow direction because the convective O_2 flux into the gas compartment was about 56% larger than the O_2 consumed in the reaction. By assuming that the O_2 concentration at the catalyst surface, $C_{\mathrm{O_2,Cat.}}$, dropped to 0 mol cm^{-3} when the current became limited, we calculated $k_{\mathrm{O_2}}$ with (S2).[2]

$$\dot{n}_{\mathrm{O_2,lim}} = k_{\mathrm{O_2}} \cdot \Delta C_{\mathrm{O_2}} = k_{\mathrm{O_2}} \cdot (C_{\mathrm{O_2,bulk}} - C_{\mathrm{O_2,Cat.}}) = k_{\mathrm{O_2}} \cdot C_{\mathrm{O_2,bulk}} \tag{S2}$$

We determined the bulk oxygen concentration, $C_{\mathrm{O_2,bulk}}$, with the ideal gas law (S3). We assumed the gas temperature was equal to the ambient temperature of $T = 20$ °C. The partial pressure of oxygen, $p_{\mathrm{O_2}}$, was calculated assuming a volumetric concentration of 21% of the recorded gas pressure, p_{G}, with (S4).[2]

$$C_{\mathrm{O_2,bulk}} = \frac{p_{\mathrm{O_2}}}{R\,T} \tag{S3}$$

$$p_{\mathrm{O_2}} = 21\% \cdot p_{\mathrm{G}} \tag{S4}$$

Finally, the overall O_2 mass transfer coefficient of the GDE, $k_{\mathrm{O_2}}$ in cm s^{-1}, can be calculated with (S5) after substituting (S4) and (S3) into equation (S2) and rearranging the factors. The random error of the mass transfer coefficient, $\sigma_{k_{\mathrm{O_2}}}$, was also calculated using with (S5) by replacing the j_{lim} with the average sample standard deviation of the limiting current density, $\sigma_{j_{\mathrm{lim}}}$.[2] The resulting $k_{\mathrm{O_2}}$ and all other numerical values of various calculation steps are listed in **Table S3**.

$$k_{\mathrm{O_2}} = \frac{C_{\mathrm{O_2,bulk}}}{\dot{n}_{\mathrm{O_2,lim}}} = \frac{21\% \cdot p_{\mathrm{G}} \cdot F \cdot z}{R \cdot T \cdot j_{\mathrm{lim}}} \tag{S5}$$

Table S3: Data processing overview for limiting overall O_2 mass transfer coefficients, k_{O_2}. The absolute pressure of the gas feed is p_G. The potential window of the limiting current density plateau is between the lower limit, $E_{lim,lower}$, and the upper limit, $E_{lim,upper}$. The limiting current density is j_{lim} and its sample standard deviation is $\sigma_{j_{lim}}$. The limiting O_2 molar flux is $\dot{n}_{O_2,lim}$. The estimated random error of the mass transfer coefficient is $\sigma_{k_{O_2}}$.

p_G	1.49 bar	1.37 bar
$E_{lim,lower}$	−1.4 V vs. SHE	−1.5 V vs. SHE
$E_{lim,upper}$	−1.2 V vs. SHE	−1.3 V vs. SHE
j_{lim}	–377 mA cm^{-2}	–464 mA cm^{-2}
$\sigma_{j_{lim}}$	±4 mA cm^{-2}	±4 mA cm^{-2}
$\dot{n}_{O_2,lim}$	0.98 10^{-6} mol cm^{-2} s^{-1}	1.13 10^{-6} mol cm^{-2} s^{-1}
$\boldsymbol{k_{O_2}}$	**0.076 cm s^{-1}**	**0.096 cm s^{-1}**
$\boldsymbol{\sigma_{k_{O_2}}}$	**±0.001 cm s^{-1}**	**±0.001 cm s^{-1}**

2.4 CO_2 electrolysis experiments

2.4.1 Engineering of the CO_2 electrolysis setup

The CO_2 reduction experiments were carried out with the electrolysis setup shown in **Figure S7**. We used Labview (Version 2018, National Instruments) to record online data of the various sensors and to control the pump and the electronic valves.

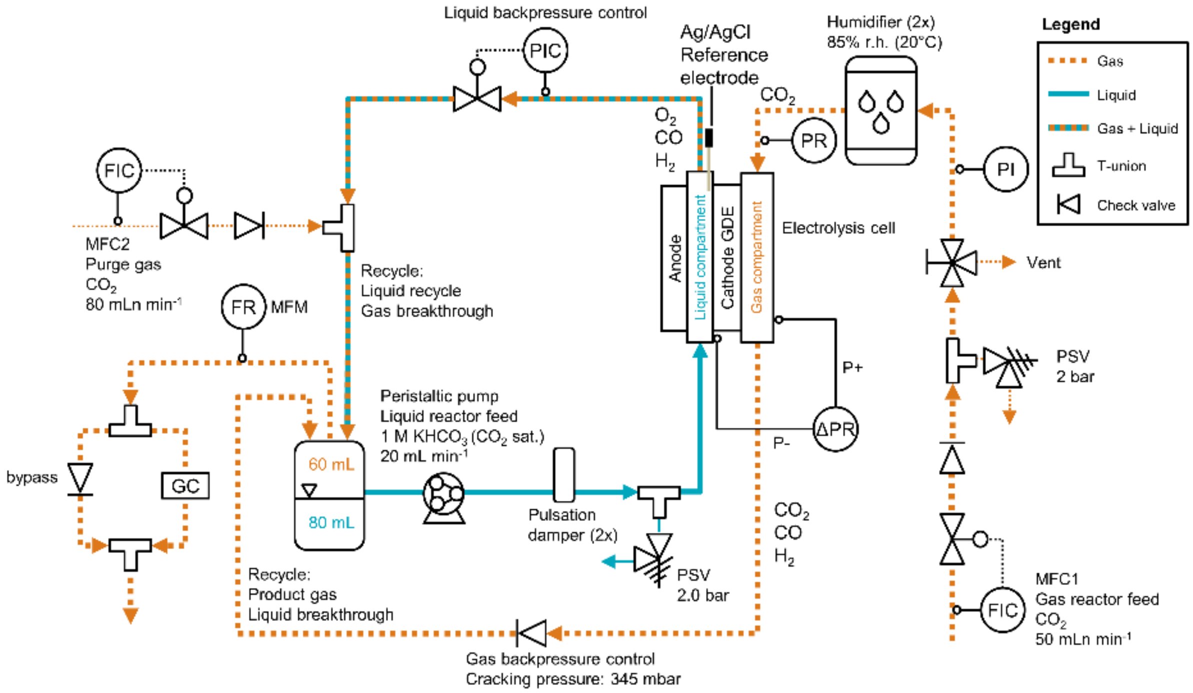

Figure S7: Extended process flow diagram for CO_2 electrolysis setup with differential pressure control. The gas flow rates were controlled with mass flow controllers (MFC). Check valves were used to prevent the backflow of liquid into the MFCs. Pressure safety valves (PSV) were installed in line to prevent the unexpected buildup of pressure. The gas feed pressure was measured with an analog pressure indicator (PI) and recorded after the humidifiers (PR). The differential pressure between the gas and the catholyte compartment was recorded with a differential pressure meter (ΔPR). The backpressure of the electrolyte stream was controlled with an electronic control valve (PIC). The product gases were collected from all process streams and combined in the head space of the electrolyte reservoir. Their combined flow rate was recorded (FR) with a mass flow meter (MFM) and the composition analyzed with a gas chromatography system (GC) to calculate the Faradaic efficiency.

Gas feed flow path

The CO_2 feed gas was supplied from a CO_2 cylinder. The gas flow rate was controlled and measured with a mass flow controller (MFC1) of the type F-201CV-500 from Bronkhorst (Netherlands). We passed the gas through two custom-made bubble columns (**Figure S8** and **Figure S9**) in series to humidify the feed with water vapor. The temperature and relative humidity of the gas feed was recorded after the humidification stage with a humidity sensor

(Type: HC2A-S Hygroclip RV+T sensor; Supplier: Acin Instrumenten, Netherlands). The pressure of the gas feed was recorded with a Deltabar S pressure meter (Endress+Hauser, Switzerland). We used another Deltabar S to record the pressure difference between the gas compartment (positive terminal: P+) and the liquid compartment (negative terminal: P-). The backpressure of the gas outlet was set by a SS-CHS2-5 check valve (Swagelok, Netherlands) with a nominal cracking pressure of 345 mbar.

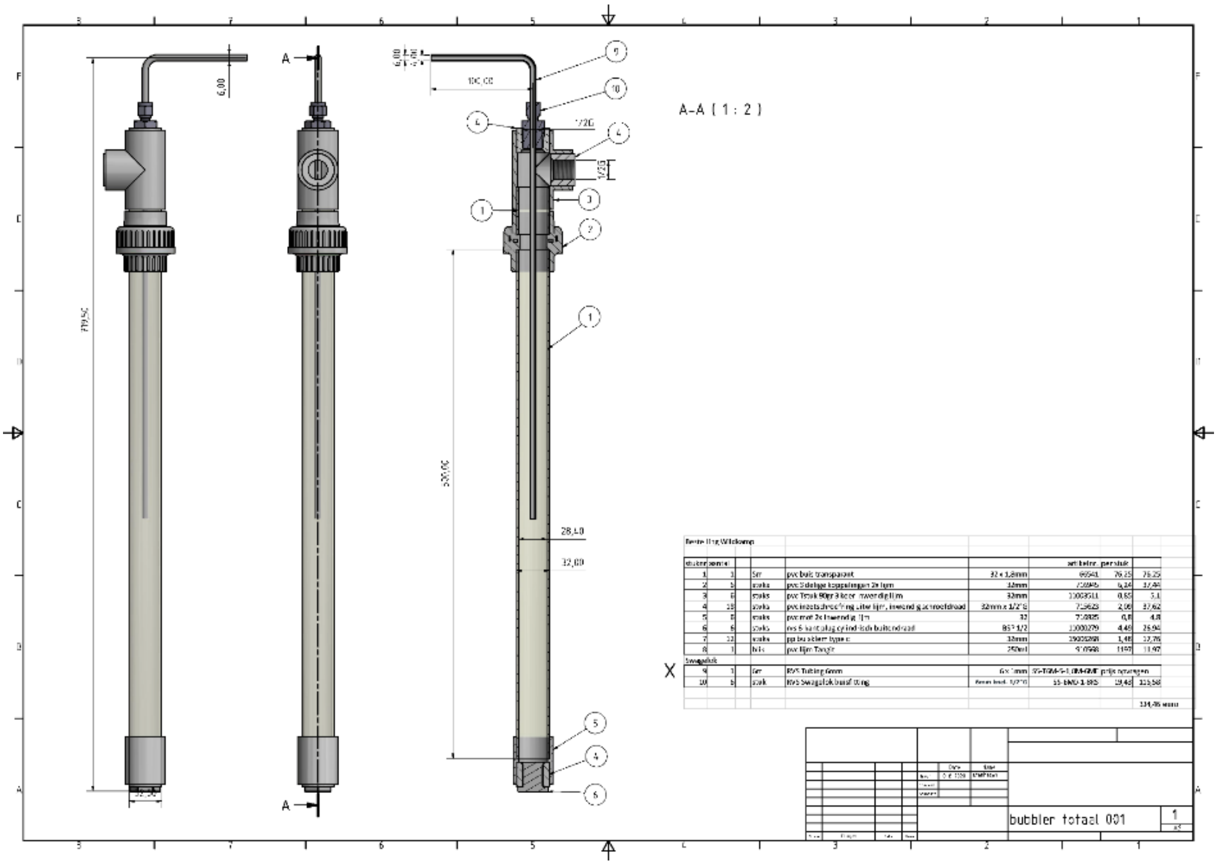

Figure S8: Technical drawing of the humidifier column. The measurement unit is mm.

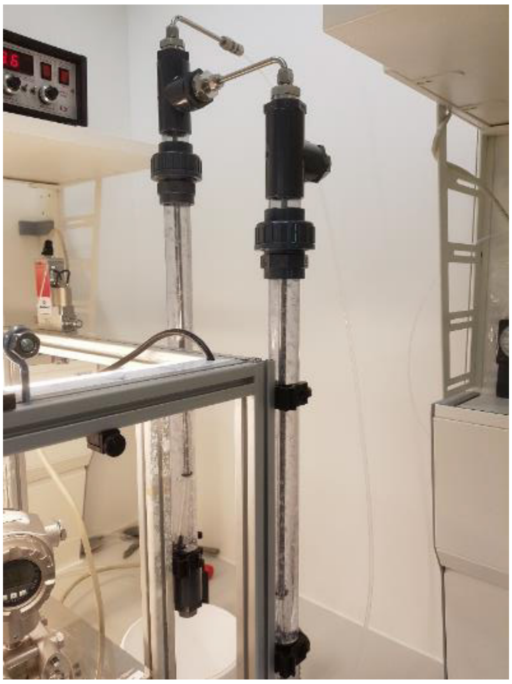

Figure S9: Two custom-made humidifier columns made from PVC pipes were used to humidify the CO_2 feed to 85 % relative humidity (r.h.) at 20°C.

Electrolyte flow path

The 1 M $KHCO_3$ electrolyte saturated with CO_2 was prepared by diluting concentrated KOH (50 wt%, analytical grade, Alfa Aesar) to 1 M KOH. The CO_2 was bubbled through the solution until the pH value was stable. The bulk pH of the electrolyte was measured prior to the experiments and is listed in the accompanying **Excel file**. The liquid lines and reactor were flushed before every experimental run. The electrolyte reservoir and liquid lines were filled with fresh electrolyte. We used a peristaltic pump (Type: Masterflex L/S peristaltic pump; Supplier: Cole Parmer) to recirculate the electrolyte through the reactor and the liquid lines with a flow rate of 20 mL min^{-1}. Two pulsation dampers (Types: FPD 1.06, FPD 1.10; Supplier: KNF, Switzerland) reduced the pressure fluctuations caused by the pump. We controlled the liquid back pressure with an electronic control valve (Type: P-502C-6K0R; Supplier: Bronkhorst, Netherlands).

Product gas flow path

Unreacted CO_2 and product gases left the reactor through the gas outlet and entered the head space of the electrolyte reservoir. Product gases forming on the catholyte side (CO, H_2) and the anode side (O_2) were carried out of the reactor by the electrolyte stream. We added a CO_2 purge gas stream to facilitate the transfer of product gases into the gas phase. The CO_2 purge gas stream further ensured that the electrolyte remained saturated with CO_2 during the experimental run. All the product gases were collected in the headspace of the electrolyte reservoir and passed through a mass flow meter (MFM) to record the flow rate (Type: F-111B-500; Supplier: Bronkhorst, Netherlands). The gas composition was analyzed with a gas chromatography system (Type: Compact GC 4.0; Supplier: Interscience, Netherlands).

Gas feed stoichiometry

The CO_2 stoichiometry factor, $\lambda_{\mathrm{CO_2}}$, is defined by the ratio of molar CO_2 flux supplied in the gas feed to the reaction rate (FE_{CO} = 100%).[7] Similarly, the H_2O stoichiometry factor, $\lambda_{\mathrm{H_2O}}$, is defined by the ratio molar H_2O vapor flux in the feed to the reaction rate.[8] The water vapor pressure in the humidifier was determined with the Antoine equation.[9] The calculations are listed in the accompanying Excel file of the SI. The resulting values are listed in **Table S4**.

Table S4: Gas feed stoichiometry listed as a function of current density, j, for an electrode area of 3.8 cm^2. The gas feed was composed of 50 mLn min^{-1} CO_2 and 0.7 mLn min^{-1} H_2O (≙ 85% relative humidity at 20°C and 1.4 bar). Normal conditions are 0°C and 1.01325 bar. The flow rate of CO at normal conditions is $\dot{V}_{\mathrm{CO}}$. The stoichiometry factors for CO_2 and H_2O are $\lambda_{\mathrm{CO_2}}$ and $\lambda_{\mathrm{H_2O}}$, respectively.

[redacted]			
**mA cm^{-2}**	mLn min^{-1}	-	-
**10**	0.26	189	2.6
**50**	1.32	38	0.5
**100**	2.65	19	0.3
**200**	5.30	9	0.1

CO_2 is supplied in large excess for all studied j (**Table S4**). According to literature, no additional benefit for FE_{CO} is gained beyond a value of $\lambda_{\mathrm{CO_2}} \geq 4$.[7, 10] This implies that the mass transfer of CO_2 from the bulk of the gas feed to the surface of the gas diffusion layer is not limiting. We selected a relatively large CO_2 feed flow rate of 50 mLn min^{-1} to shorten the equilibration time between parameter sets to 6 min (see following section).

The supplied H_2O vapor is insufficient to cover the consumption of H_2O by the CO_2R reaction for all values of j above 10 mA cm^{-2} (**Table S4**: $\lambda_{\mathrm{H_2O}}$ < 1). According to a recent study by Hoof *et al.,*[8] values of $\lambda_{\mathrm{H_2O}}$ < 1 have a detrimental effect on the stability of zero gap-type CO_2 electrolyzers. For our flowing electrolyte-type electrolyzer, we assume that the cathode remains better hydrated through the direct contact with the electrolyte. However, this might leads to a local depletion of H_2O in the electrolyte and salt formation contributing to flooding.[11, 12]. The effect of $\lambda_{\mathrm{H_2O}}$ on GDE flooding in CO_2 electrolyzers with flowing catholyte is an interesting subject for future investigations.

Calculation of Faradaic efficiency

The Faradaic efficiency of gas species FE_i (H_2, CO) was calculated with the recorded current, I, Faraday's constant, F, the stoichiometric number of electrons exchanged, z_i ($z_i = 2$ for H_2 and CO), the corrected MFM gas flux, $\dot{N}_{\mathrm{MFM}}$, and the gas concentration, C_i, using (S7).

$$FE_i = \frac{z_i \cdot F \cdot C_i \cdot \dot{N}_{\mathrm{MFM}}}{I} \quad \text{(S6)}$$

The simple gas conversion factors provided by the supplier of the MFM are listed in **Table S4**. We did not detect the components CH_4 or C_2H_4 in any of our product gas samples. Therefore, CO_2 is the only component that differs significantly from a conversion factor of 1 in our product mixture. This allows us to simplify the calculation of the mixture conversion factor K_{mix}.

Table S5: Single component gas conversion factors K_{i} for 20°C and 1 atm provided by Bronkhorst General Manual Digital Instruments.

**H_2**	1.01
**N_2**	1.00
**O_2**	0.98
**CO_2**	0.74
**CO**	1.00
**CH_4**	0.76
**C_2H_4**	0.60

We assume that the components N_2, H_2, O_2, are equivalent in their conversion factor to CO. We then developed a simple linear model using the Fluidat flow calculation tool (Bronkhorst, Netherlands). This tool allows to calculate K_{mix}, which converts the recorded gas flow, $\dot{N}_{\mathrm{MFM,nominal}}$, (MFM calibrated for 90 vol% CO_2, 5 vol% CO, 5 vol% H_2 at 10 bar (a) and 20°C) to the corrected gas flow (actual product mixture at 0.1 bar (g) and 20°C). The corrected MFM gas flux, $\dot{N}_{\mathrm{MFM}}$, is then calculated with (S8). Our model assumes the product gas mixture is a two component mixture made up of CO_2 and CO (**Figure S10**).

$$\dot{N}_{\mathrm{MFM}} = \dot{N}_{\mathrm{MFM,nominal}} \cdot K_{\mathrm{mix}} \quad \text{(S7)}$$

The mixture conversion factor, K_{mix}, is calculated with the regression formula (S9) determined in **Figure S10**.

$$K_{\mathrm{mix}} = 1.291 - 0.2764 \cdot \frac{C_{\mathrm{CO_2}}}{[\mathrm{vol\%}]} \quad \text{(S8)}$$

We calculated the volumetric concentration of CO_2, $C_{\mathrm{CO_2}}$, in vol % with (S10).

$$C_{\mathrm{CO_2}} = 100\,\mathrm{vol\%} - C_{\mathrm{CO}} - C_{\mathrm{H_2}} - C_{\mathrm{O_2}} - C_{\mathrm{N_2}} \quad \text{(S9)}$$

The resulting Faradaic efficiency values of the product gases CO and H_2 are listed in the accompanying **Excel file**.

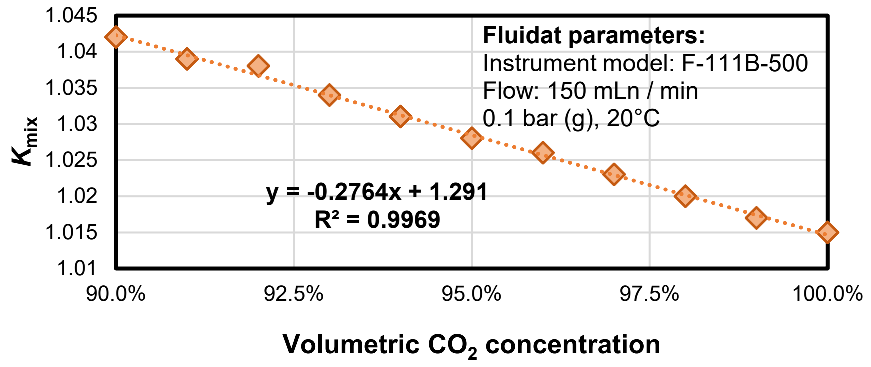

Figure S10: Determination of the mixture conversion factor, K_{mix}: The linear regression model to calculate K_{mix} is based on data points calculated with the Fluidat flow calculation tool (Bronkhorst, Netherlands). The gas mixture consists of CO_2 and CO.

2.4.2 Experimental timeline for CO_2 reduction performance with current density steps

We measured the Faradaic efficiency and cathode potential for the carbon-free GDE (Sample Ag 114-2) at three different current density steps (**Figure S11**). After installing the flow cell into the experimental setup and priming the fluid lines, we increased the liquid backpressure to achieve a flow-by regime at the GDE. We waited for 6 min after the start of each current density step (–10, –100, –200 mA cm^{-2}) so the system could reach a steady state. For the first two current densities, we collected three GC injections. We carried out additional GC injections at –200 mA cm^{-2} to assess the effect of the observed flooding. The **accompanying Excel sheet** lists the exact GC injection times. The sheet also includes all measured process parameters, such as current density or fluid flow rates, and the resulting performance metrics like the Faradaic efficiency and electrical potentials.

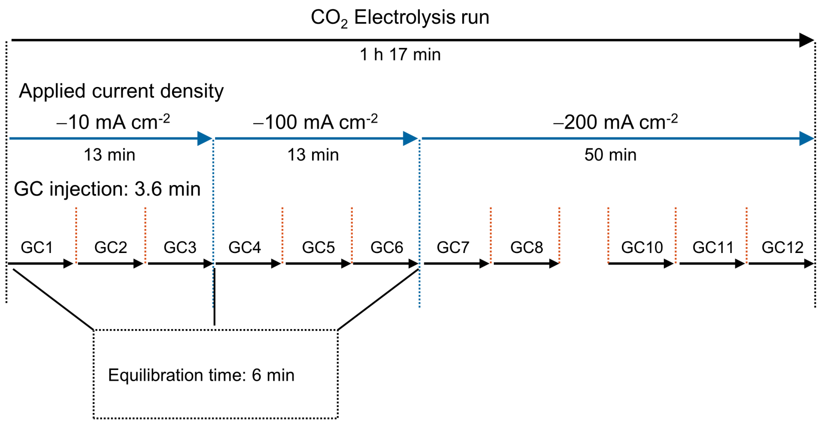

Figure S11: Experimental timeline of CO_2 electrolysis performance test with current density steps. After setting the potentiostat and balancing the pressure between liquid and gas compartment, we let the system equilibrate for 6 min. Then carried out at least three GC injections before continuing to the next current density step.

3. Supplementary results and discussion

This section presents additional results for the SEM imaging, CO_2 performance test, and assessment of chemical changes to the electrodes after electrolysis.

3.1 Microstructure investigation (SEM)

The Ag-based GDE exhibits circular patterns at low magnifications between 30x and 200x (**Figure S12**). These are created by the current collector gauze, which lies underneath the sintered coating. Larger pores with a diameter of up to 40 µm are visible in depressions at the surface, however, closer inspection revealed that these do not extend through the entire thickness of the electrode.

30x 100x 200x

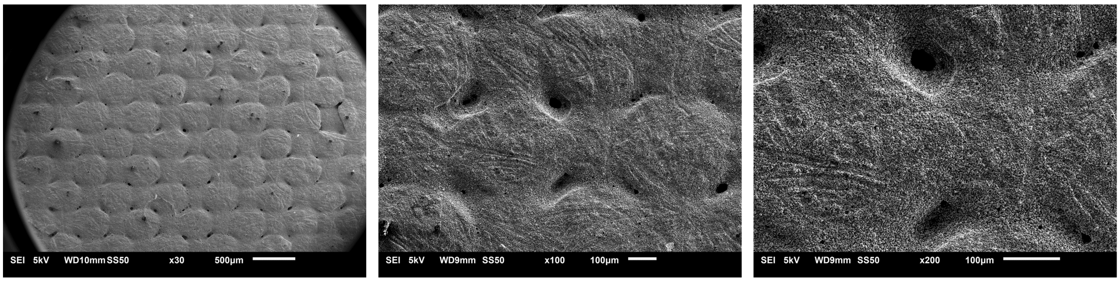

Figure S12: Structure of the carbon-free GDE. The images were recorded with the secondary electron imaging (SEI) detector of the SEM at an acceleration voltage of 5 kV at magnifications 30x, 100x, and 200x.

At magnifications between 500x and 5000x (**Figure S13**), the structure of the sintered PTFE and silver particles becomes visible. Primary silver particles have a diameter in the range of 1 – 5 µm. They are sintered together to form a porous structure, which is visualized by the light grey domains in the BEC images. The dark domains indicate the PTFE, which is dispersed over the electrode surface.

500x 1000x 5000x

SEI

BEC

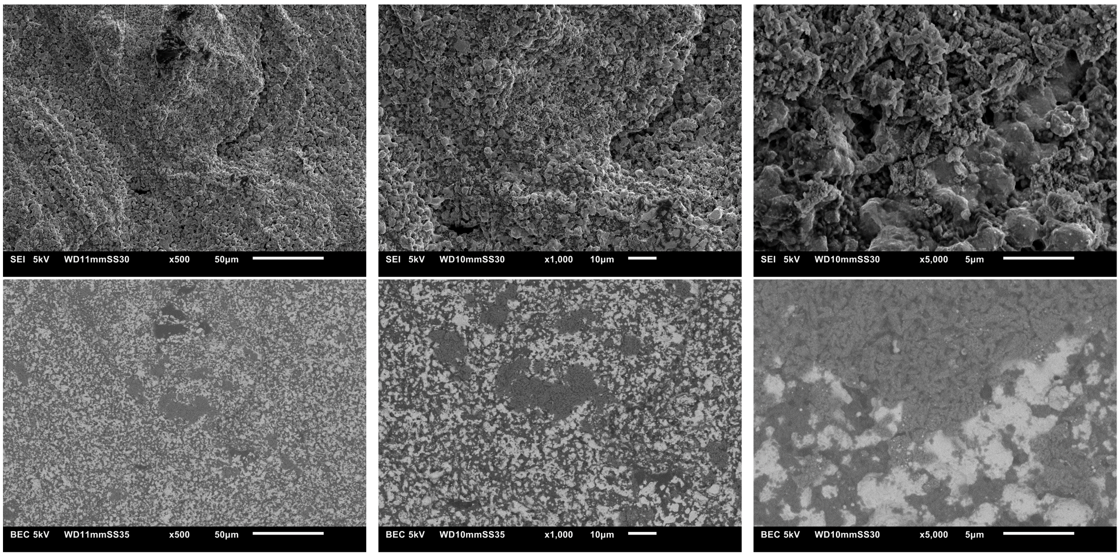

Figure S13: Structure of the Ag-based GDE's PTFE. **Top**: Secondary electron imaging (SEI) for morphology. **Bottom**: back-scattered electron composition (BEC) detector imaging of the corresponding SEI image for elemental contrast. All images were recorded with an acceleration voltage of 5 kV.

The carbon fiber substrate (CFS) of the carbon-based GDE has large pores between the PTFE-coated carbon fibers (**Figure S14**). The microporous layer (MPL) has many cracks and defects, which form during the manufacturing process. After spray coating the catalyst layer (CL) on top of the MPL, dispersed silver particles are visible at the surface. The cracks of the MPL are not filled by the coating process.

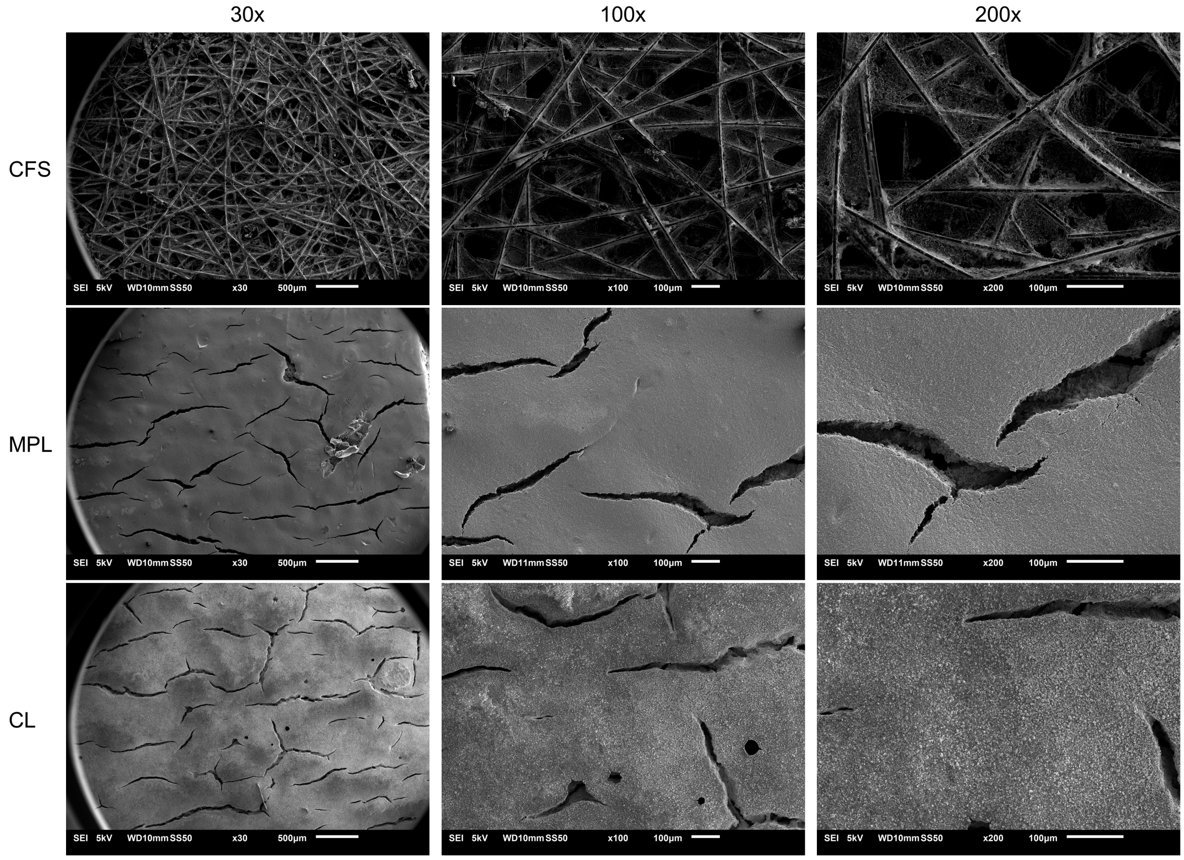

Figure S14: Morphology of the carbon-based GDE (SGL 39BC). The images of the carbon fiber substrate (CFS), the microporous layer (MPL), and the catalyst layer (CL) were recorded with the secondary electron imaging (SEI) detector of the SEM at an acceleration voltage of 5 kV at magnifications 30x, 100x, and 200x.

3.2. CO_2 reduction performance with current density steps: Flooding and salt formation

We observed the breakthrough of electrolyte during the CO_2 electrolysis experiment with sample Ag 114-2 (**Figure 4 a**). First droplets started appearing at the gas side of the GDE about 10 min after applying a current density of −200 mA cm^{-2} (−1.3 V vs. RHE) (**Figure S15 a**). Over the course of the experiment, the droplets coalesced into larger drops and started to dry out (**Figure S15 b**). After disassembling the cell, potassium (bi)carbonate salt was present at the gas side of the GDE (**Figure S15 c**).

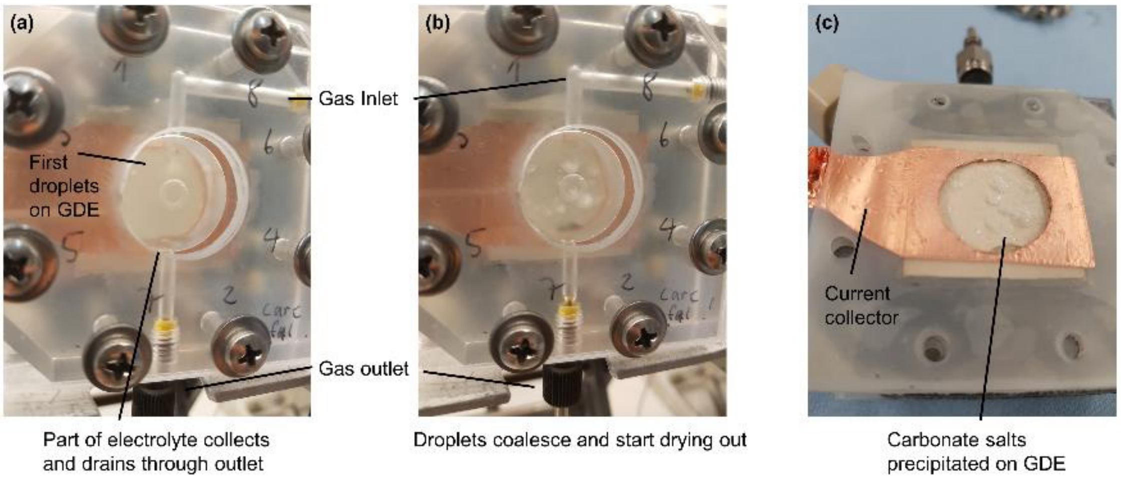

Figure S15: CO_2 electrolysis with carbon-free GDE at −200 mA cm^{-2}: electrolyte breakthrough and salt formation. **(a)** First electrolyte droplets start breaking through to the gas side of GDE. **(b)** Potassium (bi)carbonate salt starts precipitating after 40 min. **(c)** After disassembling: Gas side of GDE is covered with precipitated salt.

3.3. Post electrolysis characterization: Assessment of chemical stability

To assess the chemical stability of the carbon-free electrodes, we operated two different samples at –200 mA cm^{-2} (**Figure S16**) and –50 mA cm^{-2} (**Figure 4 b**) and applied a series of characterization methods (XRD, SEM, and XPS).

We operated a carbon-free GDE at –200 mA cm^{-2} in a gas flow-through mode (**Figure S16**). With this flow mode, we attempted to increase the mass transfer of CO_2 and reduce the saturation of the pore network through a higher gas overpressure.

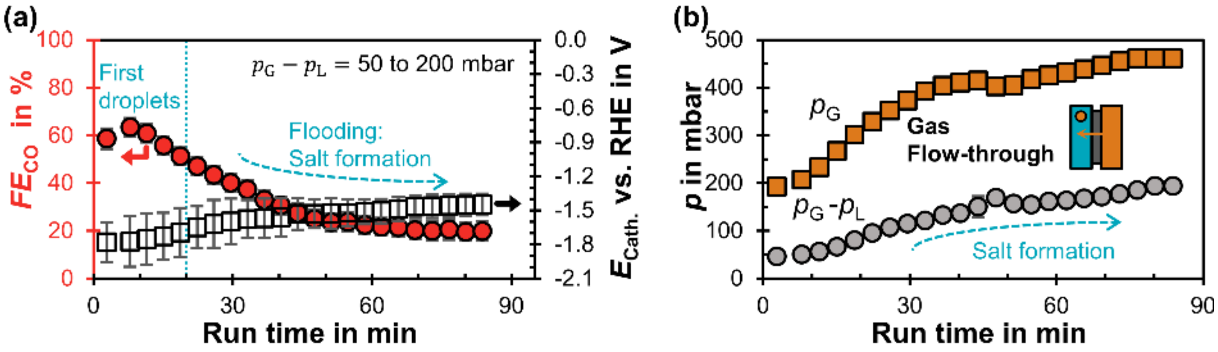

Figure S16: Chemical stability test of carbon-free GDE at –200 mA cm^{-2} in gas flow-through mode **(a)**: Faradaic efficiency for CO, FE_{CO}, as a function of run time after starting the potentiostat. The cathode potential, $E_{Cath.}$, against the reversible hydrogen electrode (RHE) is plotted on the right y-axis. The potential was corrected for the ohmic potential drop between the reference electrode and the cathode. Every data point represents a single GC injection. The error bars represent the estimated standard error. **(b)**: Gas compartment pressure, p_G, and pressure difference between gas and liquid compartment, $p_G - p_L$, increased steadily over the course of the experiment.

The development of the cathode potential, $E_{Cath.}$, is shown in **Figure S16 a**. The initial $E_{Cath.}$ is more negative compared to the flow-by mode (**Figure S16 a vs. Figure 4 a**: –1.8 V compared to –1.3 V vs. RHE) because of CO_2 bubble resistance.[3] The $E_{Cath.}$ stabilized at –1.4 V vs. RHE (**Figure S16 a**). This may be due to an increase of catalytic interface for the HER as the pores are saturated with electrolyte, which reduces the local current density and the ohmic potential losses in the electrolyte.

The flow-through mode, however, is not effective because the electrolyte still floods the GDE and starts forming salts. The increasing saturation of the porous network hinders the flow of CO_2, which raised the gas overpressure from initially 50 mbar to 200 mbar when we stopped the experiment after 84 min (**Figure S16 b)**. The gas flow-through cannot prevent flooding because electrowetting leads to strongly hydrophilic pores, which apparently would require a much higher gas pressure to drain.

3.3.1 XRD: No changes to silver bulk composition

The X-ray diffractograms before and after electrolysis consist of a single cubic Ag^0 phase (**Figure S17 a**). The reflections at 2θ = 44.6°, 51.9°, 76.6°, 93.2°, and 98.9° were attributed to the (111), (200), (220), (311), and (222) facets of Ag^0, respectively. FWHM (full width at half maximum) analysis of Ag^0 reflections suggest that Ag^0 crystalline domain size was not altered by the electrochemical treatment (**Table S5**).

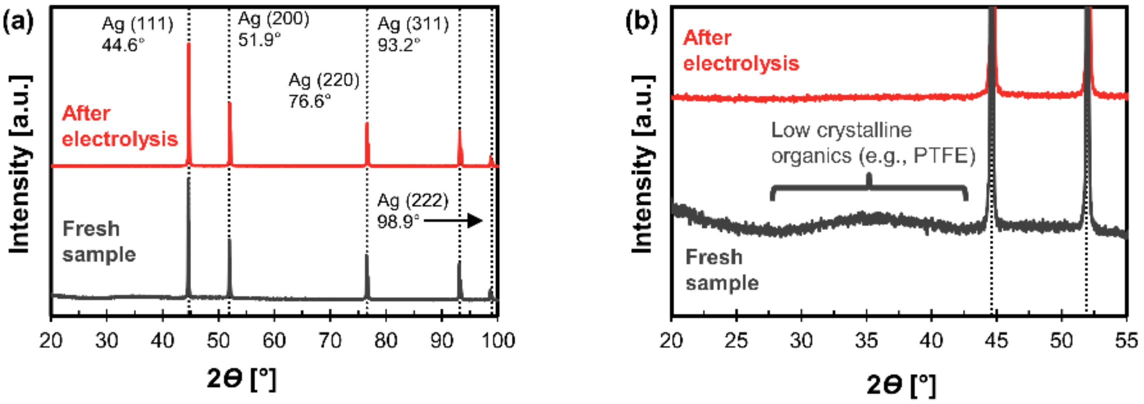

Figure S17: X-ray diffractograms for fresh carbon-free GDE) and after electrolysis at –200 mA cm^{-2}. **(a)**: Ag diffraction pattern. The Powder Diffraction File®(PDF)-2004 database of the International Centre for Diffraction Data was used for peak assignment. Both samples exhibited diffraction patterns of cubic Ag^0 (PDF #87-0720) with standard peak ratios and no variation in crystalline parameters regardless of treatment. **(b)**: Zoom-in on 2θ = 30° – 55°. Broad peak caused by low crystalline organics compound(s) (e.g., PTFE) between 30° – 40°.

Table S6: Carbon-free GDE: FWHM (full width at half maximum) analysis for fresh sample and spent sample after electrolysis at –200 mA cm^{-2}.

**Fresh sample**	0.169°	0.195°	0.265°
**After electrolysis**	0.168°	0.192°	0.252°

A small shoulder between 30° – 40°, visible in fresh GDE, became less prominent in the spent electrode (**Figure S17 b**). This peak can be attributed to low crystalline organics constituting GDEs (e.g. PTFE). No definitive assignment of PTFE peaks could be made due to the low intensity of the these reflections. We hypothesize that the disappearance of the 30° – 40° band might be indicative of PTFE degradation, which was also observed by the complementary analysis techniques (SEM, XPS).

3.3.2 SEM: PTFE surface coverage changes at high cathode overpotential

Additional SEM images recorded with the BEC detector show that GDE surface is covered with less PTFE after being operated at –200 mA cm^{-2} and $E_{\mathrm{Cath.}}$ –1.8 V vs. RHE (**Figure S18**). In contrast, the GDE, which was operated at –50 mA cm^{-2} (–1.0 V vs. RHE), looks much more similar to the surface before electrolysis.

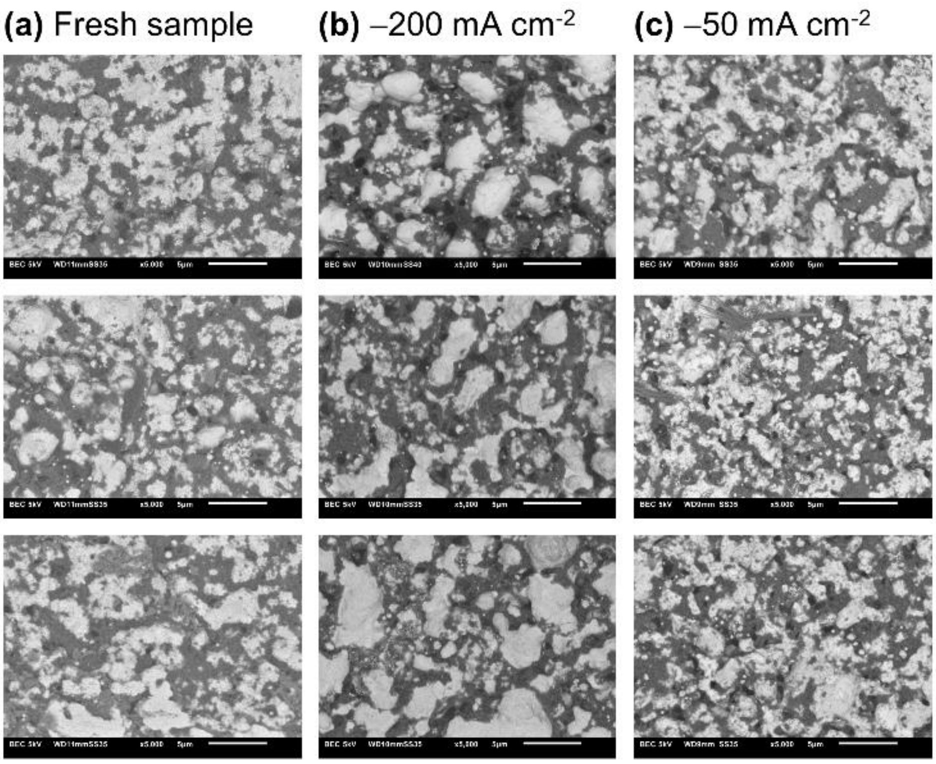

Figure S18: Surface coverage with PTFE: SEM shows the elemental contrast with images from the BEC detector (light grey domains: Ag, dark grey domains: PTFE, carbon). The length of the scale bar for a magnification of 5000x is 5 µm. **(a)**: Surface of unused sample. **(b)**: After electrolysis at –200 mA cm^{-2} for 84 min. **(c)**: After electrolysis at –50 mA cm^{-2} for 89 min at –1.0 V vs. RHE. The images of the electrolysis samples (b) and (c) were taken from the side facing the electrolyte.

3.3.3. XPS: Degradation and removal of PTFE

The surface elemental composition and chemical state of electrodes was evaluated by XPS. Bulk composition and element distribution was derived from the XPS depth profiles.

Elemental composition analysis

The surface chemical composition of GDEs was derived from wide-energy XPS spectra recorded at three separate locations for each sample. The average elemental composition and its standard deviation is summarized in **Table S6**. According to the elemental analysis, the major elements present are C, F, Ag and O, in line with expectations. Used GDE samples had impurities of K and Cu originating from the electrolyte and the copper current collector, respectively. Also, Ca, S, Si and Cl impurities were found in the miniscule amounts. Due to the strong overlap between C 1s / K 2p and Ag 3d / K 2s (**Figure S20 a**), along with a large difference in relative sensitivity factors of those spectral regions, the atomic concentrations of C, Ag and K were adjusted based on the corresponding core-level spectra.

Table S7: Elemental composition for carbon-free GDEs. **(a)** Fresh GDE sample. **(b)** After electrolysis at –200 mA cm^{-2} for 84 min with at –1.8 V vs. RHE. **(c)** After electrolysis at –50 mA cm^{-2} for 89 min with cathode potential of –1.0 V vs. RHE. For the electrolysis samples (b) and (c), the analyzed areas were facing the electrolyte during electrolysis. The average elemental concentration ± the standard error was determined from three analysis locations per sample.

**Ag**	3.8 ± 0.05 at.%	3.7 ± 0.60 at.%	5.9 ± 0.27 at.%
**C**	36.3 ± 0.40 at.%	48.7 ± 1.07 at.%	35.4 ± 0.43 at.%
**F**	57.8 ± 0.48 at.%	40.1 ± 1.08 at.%	51.3 ± 0.27 at.%
**Cu**	n.a.	0.2 ± 0.05 at.%	0.2 ± 0.02 at.%
**O**	1.2 ± 0.08 at.%	5.8 ± 0.45 at.%	3.5 ± 0.20 at.%
**Ca**	0.4 ± 0.03 at.%	0.1 ± 0.004 at.%	n.a.
**S**	0.3 ± 0.03 at.%	n.a.	n.a.
**Si**	n.a.	0.8 ± 0.05 at.%	0.9 ± 0.06 at.%
**Cl**	0.2 ± 0.01 at.%	n.a.	n.a.
**K**	n.a.	0.7 ± 0.10 at.%	2.6 ± 0.19 at.%

Ag reference foil

Sputter-cleaned Ag foil reference was used to derive the intrinsic asymmetry of Ag^0 peaks in the Ag 3d region (**Figure S19 a**). The foil was cleaned in-situ by repeated high-energy ion beam sputtering until complete disappearance of O 1s peaks. Ag oxidation state was confirmed by the modified Auger parameter α = 726.1 eV (**Figure S19 b**). The Ag $3d_{5/2}$ component of the sputter-cleaned foil was adapted as a line shape for Ag $3d_{3/2}$ component with a set of constrains listed in **Table S7**.

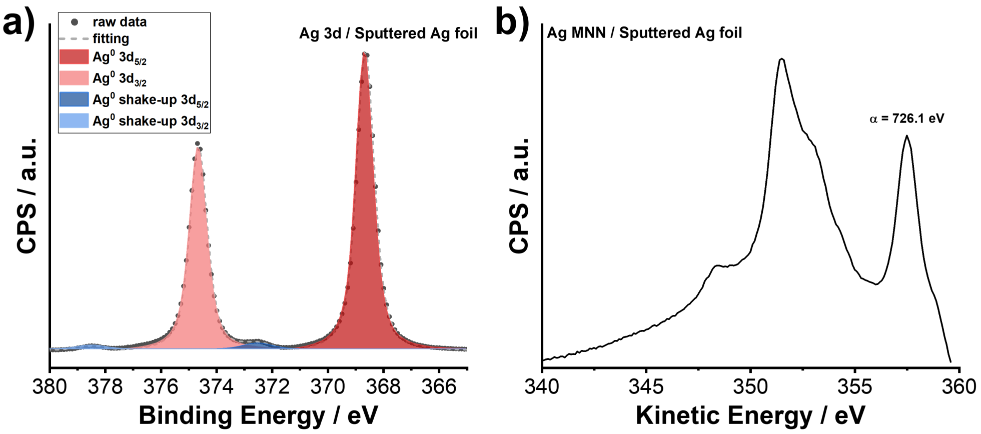

Figure S19: **(a)**: Ag 3d and **(b)**: Ag MMN XPS spectra of Ar^+-sputtered Ag reference foil.

Table S8: XPS data processing: Ag 3d fitting parameters of Ag^0 reference foil. Indices at parenthesis refer to the maximum allowed deviation (eV) in the constrain.

**Ag^0 $3d_{5/2}$**	Ag^0 ref $3d_{5/2}$	$(368.5)_{0.5}$ eV	$(1.0)_{0.2}$ eV	n/a
**Ag^0 $3d_{3/2}$**	Ag^0 ref $3d_{5/2}$	Ag^0 $3d_{5/2}$ + 6 eV	Ag^0 $3d_{5/2}$	$3d_{5/2}$ * 0.68
**Ag^0 sh-up $3d_{5/2}$**	GL(30)	Ag^0 $3d_{5/2}$ + $(3.8)_{0.2}$ eV	$(Ag^0\ 3d_{5/2} * 1.2)_{0.2}$	n/a
**Ag^0 sh-up $3d_{3/2}$**	GL(30)	Ag^0 sh-up $3d_{5/2}$ + 6 eV	Ag^0 sh-up $3d_{5/2}$	$3d_{5/2}$ * 0.68
**Ag_2O $3d_{5/2}$**	GL(30)	Ag^0 $3d_{5/2}$ – $(0.8)_{0.2}$ eV	$(1.5)_{0.2}$ eV	n/a
**Ag_2O $3d_{3/2}$**	GL(30)	Ag_2O $3d_{5/2}$ + 6 eV	Ag_2O $3d_{5/2}$	$3d_{5/2}$ * 0.68

Carbon-free GDE: Chemical state of silver

Ag 3d spectra of fresh and spent carbon-free GDEs suggested that silver is mostly present in its metallic form, yielding asymmetric peaks with the binding energy, *BE*, of $BE_{5/2}$ = 368.2 ± 0.1 eV (**Figure S20 a**).

This assignment is supported by the values of modified Auger parameters (α = 725.9 ± 0.1 eV), characteristic for Ag^0 (**Figure S20 b**).[13] A presence of native Ag_2O was evident in the fresh GDE sample (**Figure S20 b**), which can be seen as a second component in the surface-sensitive region of Ag MNN (α_2 = 724.4 ± 0.1 eV). However, the fraction of Ag_2O was not affected by the electrolysis significantly, accounting for 2 – 6% of all silver in fresh and spent GDEs. A small component at *BE* ≈ 377 eV in spent samples was assigned to the overlapping K 2s spectral region, originating from the residual electrolyte.

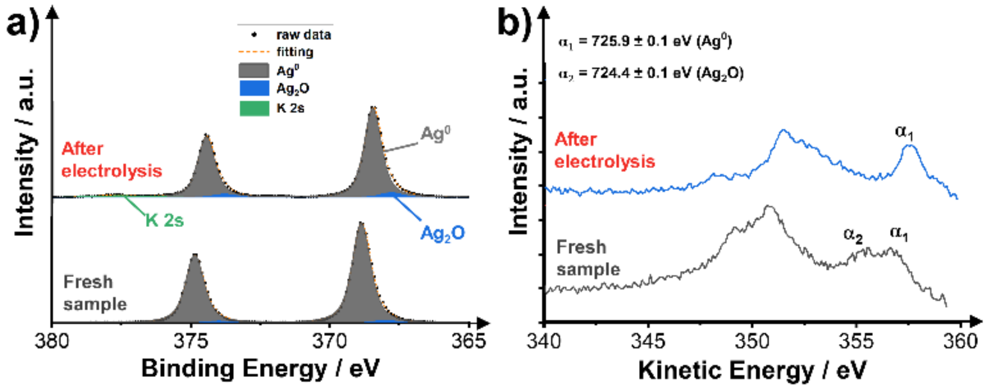

Figure S20: **(a)** Ag 3d and **(b)** Ag MNN XPS spectra of carbon-free GDE before and after electrolysis at –200 mA cm^{-2}.

Carbon-free GDE: Chemical state of carbon

C 1s XPS spectra were used to observe the change in carbon bonds distribution. C 1s spectrum of fresh Ag-GDE is primarily composed of CR (C-C, C-H) and CF_2 peaks with *BE* of 284.8 eV and 291.8 eV, respectively (**Figure S21**). A spectral region between 286 eV and 290 eV features multiple minor peaks which can be assigned to various forms of oxygenated carbon (COR, C=O, COOR).

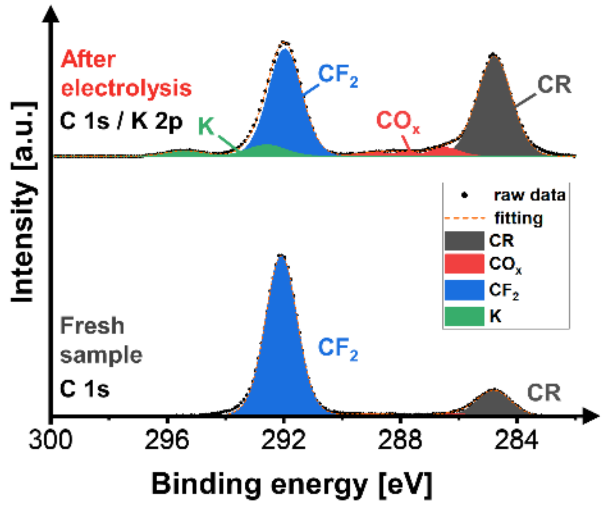

Figure S21: Chemical state of carbon before and after electrolysis at –200 mA cm^{-2}: XPS spectrum in C 1s region (overlapping with K 2p). The K is present in residual amounts after rinsing the $KHOC_3$ electrolyte off after electrolysis.

The relative amount of CF_2 bonds was strongly reduced by electrolysis (**Table S8**). The shift to CR and CO_x bonds is especially significant for the sample that was subjected to j = –200 mA cm^{-2} (–1.8 V vs. RHE). As we know from **Table S6**, the used samples exhibit an increased oxygen concentration relative to the fresh sample (4 – 6 at.% vs. 1 at.%.) As Ag was not oxidized significantly during electrolysis (**Figure S20**), the additional oxygen is present in the form additional CO_x bonds, which probably originate from residual $KHCO_3$ electrolyte salts (**Table S8**).

Table S9: Relative fraction of carbon bond types: carbon–fluorine bonds (CF_2), which are present in PTFE, carbon–oxygen bonds (CO_x: COR, CO, or COOR), and saturated C–R bonds like C–C or C–H as a share of all carbon bonds C–X. **(a)** Fresh GDE sample. **(b)** After electrolysis at –200 mA cm^{-2} for 84 min (–1.8 V vs. RHE). **(c)** After electrolysis at –50 mA cm^{-2} for 89 min (–1.0 V vs. RHE). For the electrolysis samples (b) and (c), the analyzed areas were facing the electrolyte during electrolysis.

**CF_2 / C–X**	84 ± 1.6%	45 ± 1.3%	76 ± 0.6%
**CO_x / C–X**	4 ± 0.4%	7 ± 0.5%	4.0 ± 0.1%
**CR / C–X**	13 ± 1.2%	48 ± 1.2%	20%

Depth profile analysis

The XPS depth profiles of the carbon-free GDE shows a significant change in chemical composition after electrolysis at –200 mA cm^{-2} with an initial cathode potential of –1.8 V vs. RHE (**Figure S22 a** – **c**). In contrast, the carbon-free GDE operated at –50 mA cm^{-2} (–1.0 V vs. RHE) shows no significant decrease in F or increase in Ag compared to the fresh sample (**Figure S22 d** – **f**). We note that the concentration for the –50 mA cm^{-2} experiment (**Figure S22 d** – **f**) are more homogeneous over the depth profile compared to the fresh sample (**Figure S22 a** – **c**). The reason for this deviation might be that the profiles were recorded from two different sample batches (–200 mA cm^{-2} experiment: Ag 114 -3 and –50 mA cm^{-2} experiment: Ag 178-1).

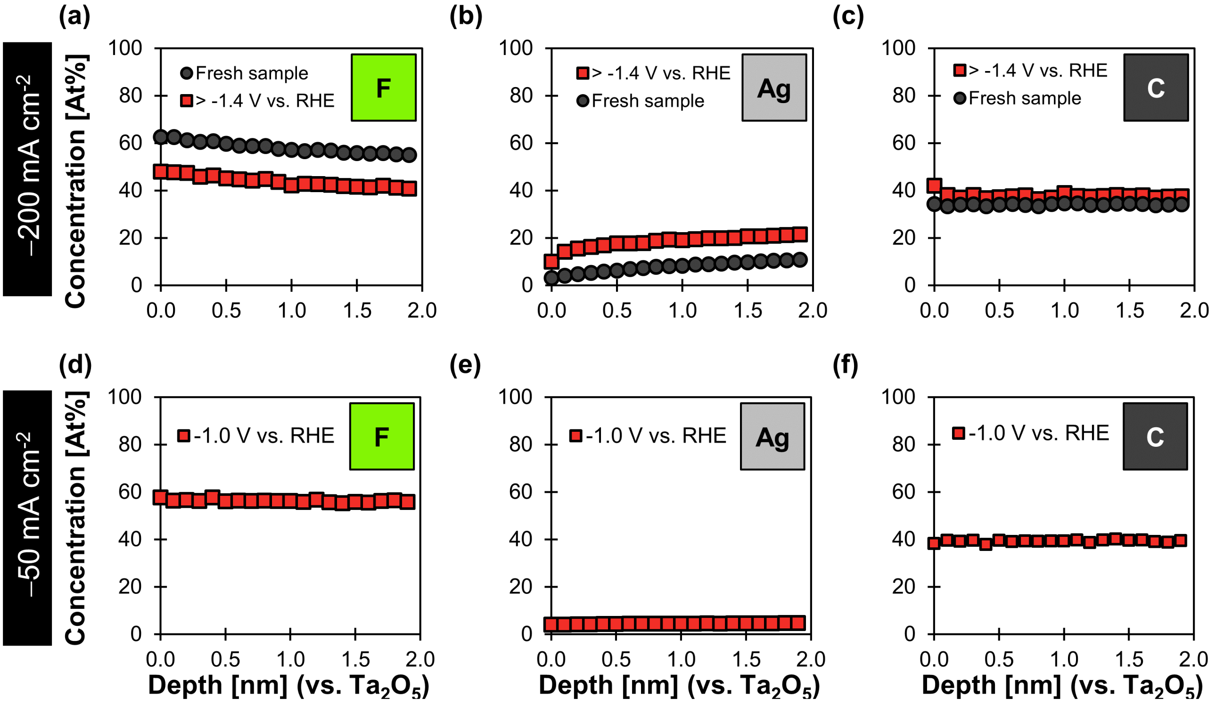

Figure S22: XPS depth profiles of carbon-free GDEs. The x-axis shows the depth profile calibrated against a Ta_2O_5 standard sputtered with Ar^+ ions. The y-axis shows the relative atomic concentrations of F, Ag, and C (other elements were not measured in this measurement mode). **(a), (b)**, **(c)**: Fresh sample and sample after electrolysis at –200 mA cm^{-2} for 84 min (–1.8 V vs. RHE). **(d)**, **(e)**, **(f)**: After electrolysis at –50 mA cm^{-2} for 89 min (–1.0 V vs. RHE).

These findings suggest that PTFE degrades and/or is removed from the GDE surface. The homogeneous loss of F over the profile is in alignment with the reductive elimination mechanism proposed by Shapoval *et al.*.[14] According to this mechanism, F$^-$ is eliminated below a cathodic potential of –1.3 V vs. RHE and carbonaceous degradation products are left behind (**Figure S23**).

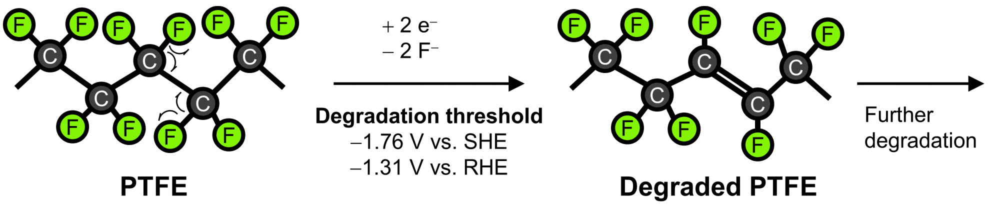

Figure S23: Cathodic reduction of PTFE: Shapoval *et al.*.[14] proposed that C–F bonds in sterically stressed sections of the polymer are susceptible to reduction and elimination of the fluorine atom. They reported a degradation threshold of −1.76 V vs. SHE, which corresponds to a cathode potential of −1.31 V vs. RHE in 1 M $KHCO_3$ (pH = 7.56).

References

1. Franzen, D., B. Ellendorff, M.C. Paulisch, A. Hilger, M. Osenberg, I. Manke, and T. Turek, *Influence of Binder Content in Silver-Based Gas Diffusion Electrodes on Pore System and Electrochemical Performance.* Journal of Applied Electrochemistry, 2019. **49**(7): p. 705-713.
2. Baumgartner, L.M., C.I. Koopman, A. Forner-Cuenca, and D.A. Vermaas, *Narrow Pressure Stability Window of Gas Diffusion Electrodes Limits the Scale-up of Co2 Electrolyzers.* ACS Sustainable Chemistry & Engineering, 2022. **10**(14): p. 4683-4693.
3. Duarte, M., B. De Mot, J. Hereijgers, and T. Breugelmans, *Electrochemical Reduction of Co2: Effect of Convective Co2 Supply in Gas Diffusion Electrodes.* ChemElectroChem, 2019. **6**(22): p. 5596-5602.
4. Endrődi, B., G. Bencsik, F. Darvas, R. Jones, K. Rajeshwar, and C. Janáky, *Continuous-Flow Electroreduction of Carbon Dioxide.* Progress in Energy and Combustion Science, 2017. **62**: p. 133-154.
5. Baumgartner, L.M., C.I. Koopman, A. Forner-Cuenca, and D.A. Vermaas, *When Flooding Is Not Catastrophic – Woven Gas Diffusion Electrodes Enable Stable Co2 Electrolysis.* ACS Applied Energy Materials, 2022(5): p. 15125-15135.
6. Zeng, Z. and R. Grigg, *A Criterion for Non-Darcy Flow in Porous Media.* Transport in Porous Media, 2006. **63**(1): p. 57-69.
7. Jeanty, P., C. Scherer, E. Magori, K. Wiesner-Fleischer, O. Hinrichsen, and M. Fleischer, *Upscaling and Continuous Operation of Electrochemical Co2 to Co Conversion in Aqueous Solutions on Silver Gas Diffusion Electrodes.* Journal of CO2 Utilization, 2018. **24**: p. 454-462.
8. Hoof, L., K. Pellumbi, S. Heuser, D. Siegmund, K. junge Puring, and U.-P. Apfel, *Water Management as a Key Parameter for Scaling up a Co2 Electrolyzer.* Chemie Ingenieur Technik, 2023.
9. Tosun, İ., *The Thermodynamics of Phase and Reaction Equilibria*. 2021: Elsevier.
10. Haas, T., R. Krause, R. Weber, M. Demler, and G. Schmid, *Technical Photosynthesis Involving Co2 Electrolysis and Fermentation.* Nature Catalysis, 2018. **1**(1): p. 32-39.
11. Leonard, M.E., L.E. Clarke, A. Forner-Cuenca, S.M. Brown, and F.R. Brushett, *Investigating Electrode Flooding in a Flowing Electrolyte, Gas-Fed Carbon Dioxide Electrolyzer.* ChemSusChem, 2019. **13**(2): p. 400-411.
12. Nwabara, U.O., E.R. Cofell, S. Verma, E. Negro, and P.J.A. Kenis, *Durable Cathodes and Electrolyzers for the Efficient Aqueous Electrochemical Reduction of Co2.* ChemSusChem, 2020. **13**(5): p. 855-875.
13. Powell, C.J., *Recommended Auger Parameters for 42 Elemental Solids.* Journal of Electron Spectroscopy and Related Phenomena, 2012. **185**(1): p. 1-3.
14. Shapoval, G., A. Tomilov, A. Pud, and V. Vonsyatskii, *Electrochemical Reductive Destruction of Polytetrafluoroethylene.* Theoretical and Experimental Chemistry, 1984. **20**(2): p. 234-236.
